# An *in silico* micro-multiphysics agent-based approach for simulating bone regeneration in a mouse femur defect model

**DOI:** 10.3389/fbioe.2023.1289127

**Published:** 2023-12-14

**Authors:** Jack J. Kendall, Charles Ledoux, Francisco C. Marques, Daniele Boaretti, Friederike A. Schulte, Elise F. Morgan, Ralph Müller

**Affiliations:** ^1^ Institute for Biomechanics, ETH Zurich, Zurich, Switzerland; ^2^ Center for Multiscale and Translational Mechanobiology, Boston University, Boston, MA, United States

**Keywords:** computational modelling, agent-based model, bone healing, osteogenesis, angiogenesis, *in silico*, *In vivo*, C57Bl/6

## Abstract

Bone defects represent a challenging clinical problem as they can lead to non-union. *In silico* models are well suited to study bone regeneration under varying conditions by linking both cellular and systems scales. This paper presents an *in silico* micro-multiphysics agent-based (micro-MPA) model for bone regeneration following an osteotomy. The model includes vasculature, bone, and immune cells, as well as their interaction with the local environment. The model was calibrated by time-lapsed micro-computed tomography data of femoral osteotomies in C57Bl/6J mice, and the differences between predicted bone volume fractions and the longitudinal *in vivo* measurements were quantitatively evaluated using root mean square error (RMSE). The model performed well in simulating bone regeneration across the osteotomy gap, with no difference (5.5% RMSE, *p* = 0.68) between the *in silico* and *in vivo* groups for the 5-week healing period – from the inflammatory phase to the remodelling phase – in the volume spanning the osteotomy gap. Overall, the proposed micro-MPA model was able to simulate the influence of the local mechanical environment on bone regeneration, and both this environment and cytokine concentrations were found to be key factors in promoting bone regeneration. Further, the validated model matched clinical observations that larger gap sizes correlate with worse healing outcomes and ultimately simulated non-union. This model could help design and guide future experimental studies in bone repair, by identifying which are the most critical *in vivo* experiments to perform.

## 1 Introduction

Bone is one of the few tissues that can fully regenerate after an injury (Marsell and Einhorn, 2011). During healing, bone remodelling is responsible for a scarless regeneration that involves three key processes: tissue formation, tissue differentiation, and tissue resorption (Einhorn and Gerstenfeld, 2015). In hypertrophic non-unions, characterized by robust callus formation and effective vascularization, the healing process is hampered by inadequate mechanical stability (Wildemann et al., 2021). Mechanical loading plays a pivotal role in these processes, directing and stimulating osteogenic cells for a hypertrophic healing response supported by vasculature growth (Bahney et al., 2015; Liu et al., 2019). Conversely, in atrophic non-unions, impaired biological potential leads to limited callus formation and hindered healing, emphasizing the critical role of mechanical stability and vascular invasion in facilitating nutrient and cell transport to the injury site (Wildemann et al., 2021). Therefore, understanding how microenvironmental cues such as mechanical strain and cellular signalling regulate bone regeneration and the growth of blood vessels can provide valuable insights into the pathways that are responsible for restoring function following injury. Cross-sectional and longitudinal experimental studies have shown correlations between tissue types and micromechanical environment during healing but are limited in their ability to investigate processes and regulatory pathways (Morgan et al., 2014; Wehrle et al., 2019). Cross-sectional studies of bone regeneration are unable to track changes in protein expression or cell populations over time for an individual sample (Morgan et al., 2012; Liu et al., 2019). Longitudinal studies on the other hand can track changes in bone density over time for an individual sample but are very limited in providing data on individual cells or even soft tissues, particularly throughout the full thickness of the callus (Wehrle et al., 2019; Tourolle né Betts et al., 2020). As a complement to experimental studies, computational studies can be conducted by designing *in silico* models based on phenomenological and cellular processes.

Early computational studies identified mechano-regulation algorithms of tissue differentiation using finite element (FE) analysis to estimate the mechanical microenvironment during fracture healing. Two-dimensional models by Carter et al. (1998), Claes and Heigele (1999), Lacroix and Prendergast (2002), and Prendergast et al. (1997) predicted important aspects of tissue differentiation and maturation during bone regeneration, using “rules” based on mechanical signals such as strain, pressure, and fluid flow (Isaksson et al., 2006). Although Lacroix and Prendergast’s (2002) model also included tissue resorption, that model was only able to capture periosteal resorption and not endosteal resorption within the marrow cavity. Byrne et al. (2011) were able to simulate a fully regenerated diaphysis by adapting the mechano-regulation algorithm (Lacroix and Prendergast, 2002) to include bone resorption within the marrow cavity in a three-dimensional model. Also using FE analysis, Vetter et al. (2012) proposed a model based on thresholds for mechanical strain associated with tissue differentiation and maturation. Repp et al. (2015) expanded the model by Vetter et al. to include bone resorption in late phases of regeneration, which resulted in the cortex to be restored to its intact shape. These FE studies have laid the foundation for identifying rules pertaining to tissue differentiation and resorption; however, they are suited only for phenomenological modelling of the bone regeneration process. Specifically, the existing FE modelling approaches were unable to directly simulate cellular interactions and processes, and they stopped short of simulating remodelling. To achieve this, FE elements which were removed via resorption would have to be added again to increase the tissue modulus, a feature typically not included in FE models (Byrne et al., 2011; Repp et al., 2015).

With advances in computational power and memory, agent-based models (ABM) have grown favourable in the realm of computational biology since they allow discrete modelling of cells. As a result of many cells acting with simple rules, a system can become complex – a phenomenon known as emergence, which ABMs can simulate. In an ABM, each cell is represented by an agent that has its own state and set of rules defining its interactions with its surrounding microenvironment and other cells. Checa and Prendergast (2009) leveraged a mechanobiological ABM to simulate development of vascular networks via angiogenesis and tissue growth during bone healing under different loading and cell seeding conditions. The simulations produced spatially heterogeneous patterns of tissue differentiation, due to the morphology of the vascular networks, similar to those found in experimental studies. The revascularisation algorithm of Checa and Prendergast (2009) was incorporated by OReilly et al. (2016) into their own mechanobiological ABM to simulate the effect of disrupted angiogenesis on endochondral ossification during bone regeneration. Their model provided additional evidence that the presence of vasculature and thus sufficient oxygen availability regulates the fate of skeletal stem and progenitor cells in favour of the osteogenic lineage (Bahney et al., 2015). However, the mechanisms at the cellular and tissue scale that link osteogenesis and angiogenesis, known as osteo-angio coupling, are still incompletely understood (Morgan et al., 2012; Liu and Castillo, 2018).

Moreover, the ABM developed by Checa and Prendergast (2009) also incorporated bone resorption as a result of using previously described mechano-regulation rules (Lacroix and Prendergast, 2002) based on shear strain and fluid velocity. The bone resorption stimulus threshold was used to determine the termination of the simulations as the model did not include bone remodelling. Borgiani et al. (2021), using the same mechano-regulation rules, implemented the resorption of cartilage, fibrous tissue, and bone in their ABM of bone regeneration. Tissue resorption was not reported in the study, nor were there any considerable resorption volumes identifiable from the results (Borgiani et al., 2021). In contrast, Jaber et al. (2022) reported the relative bone resorption signal predicted from the applied mechanical loading and successfully demonstrated resorption of volumes of bone experiencing low strain. Unfortunately, the maximum theoretical bone resorption rate (BRR 0.17%/day) predicted by the applied mechanical loading was not representative of *in vivo* BRR observed via micro-computed tomography (micro-CT) during the final bone remodelling phase of bone regeneration (0.52% ± 0.25%/day) (Wehrle et al., 2019). Notwithstanding this limitation, ABMs are indeed capable of bone resorption, in addition to tissue formation and differentiation. These three essential processes should be at the heart of bone regeneration models if they aim to simulate the entire regeneration process starting with inflammation, callus formation, and finishing with remodelling of the callus. To our knowledge, however, no model of bone regeneration has captured bone remodelling of either the fracture callus or surrounding bone during the final stages of fracture healing.

In this paper, we present an *in silico* micro-multiphysics ABM which simulates remodelling during bone regeneration by incorporating the key processes of tissue formation, differentiation, resorption, and revascularisation. The *in silico* micro-multiphysics agent-based (micro-MPA) model is an ABM in which agents emulate single-cell behaviour and interact with multiple physical aspects of the microenvironment, defined by cytokine concentrations, oxygen tension, mechanical strain, and tissue mineralisation. The proposed micro-MPA model was based on the initial framework described by Tourolle (2019) that included both skeletal and immune cells with their respective paracrine signalling. This initial implementation was mainly concerned with development of the callus as a result of osteoblast polarisation. Adaptions of this framework successfully simulated homeostatic bone remodelling in ageing mouse populations (Boaretti et al., 2023) and human biopsies (Tourolle et al., 2021). By focusing on bone regeneration, we adapted the framework to include revascularisation and the remodelling of the fracture callus via resorption and *de novo* tissue formation. To validate the extended model, *in vivo* time-lapsed micro-CT data of femoral osteotomies in mice were used as the ground truth (Wehrle et al., 2019). The aim of this study was to quantitatively assess the ability of the micro-MPA model to simulate inflammation, callus formation, and finally bone remodelling within the context of bone regeneration. Then, the proposed micro-MPA model’s sensitivity to varying *in silico* osteotomy gap sizes was demonstrated by reporting the healing outcomes. We hypothesise that the proposed *in silico* model, which was calibrated to simulate successful healing, can simulate a non-union as a result of a critical osteotomy gap size.

## 2 Materials and methods

### 2.1 *In vivo* input data

**FIGURE 1:**
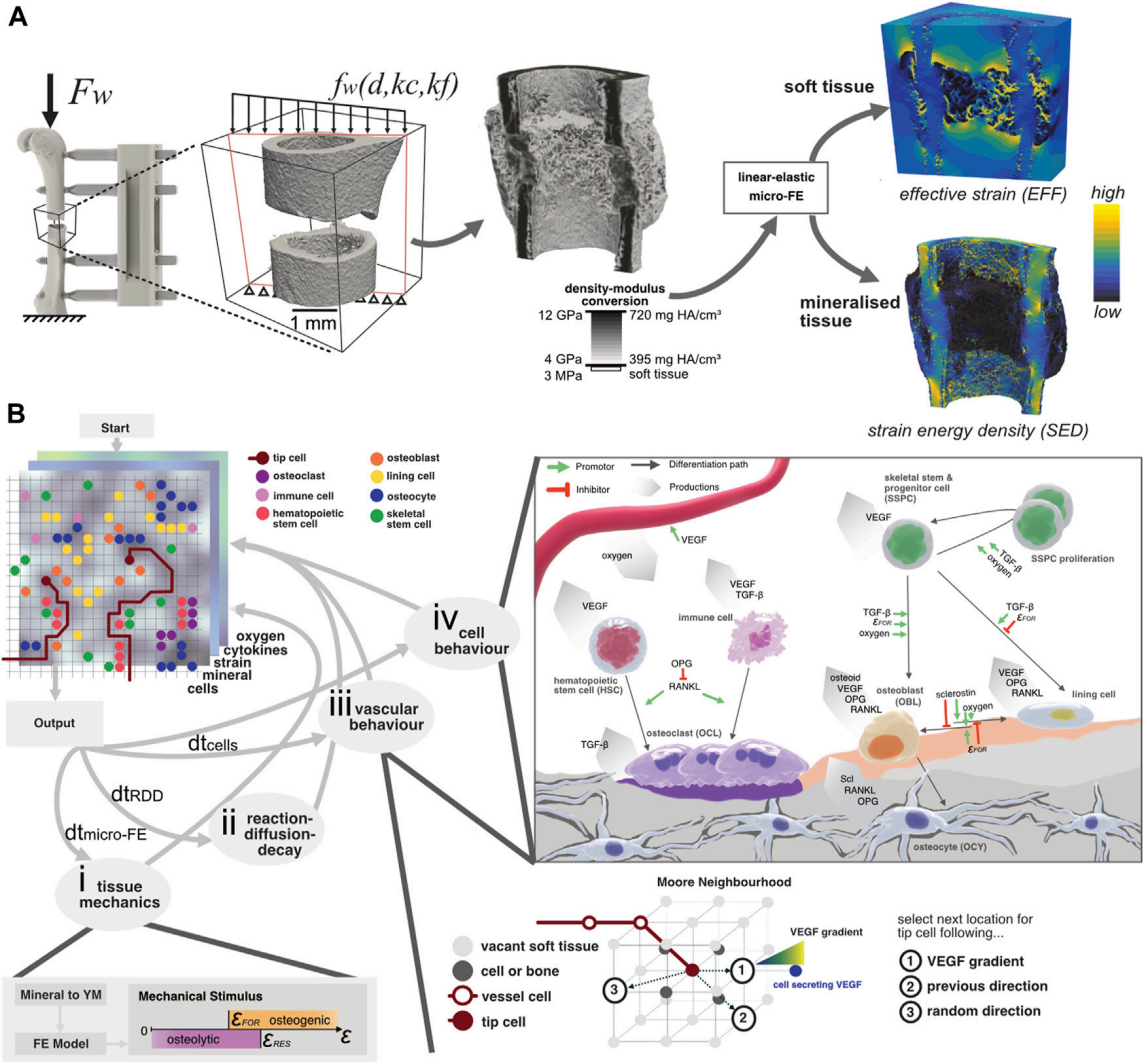
**(A)** Configuration of the loading setup where the global peak force F_w_ from ambulatory loading is applied to the femur. Due to the presence of the external fixator, the distributed load f_w_ across the osteotomy gap depends on the distance to the external fixator d, the callus stiffness kc, and the fixator’s stiffness kf. By converting the mineral density to Young’s modulus and assigning it to a “voxel” mesh, the micro-FE model is generated. Two outputs are generated: the effective strain (EFF) distribution and the strain-energy density (SED) which are used to model the strain magnitude within soft tissue and bone, respectively. **(B)** Illustration of the micro-MPA model’s simulation pipeline. The three-dimensional lattice contains many properties, including cells, mechanical signal, mineral, cytokines, and oxygen concentration. (i) The mechanical signal is updated by converting the mineral density to Young’s modulus which is given as input for the micro-FE analysis. (ii) The cytokine concentrations are solved iteratively using a reaction-diffusion-decay paradigm which includes receptor-ligand kinetics. (iii) The vascular network grows either via chemotaxis by following the VEGF gradient, continuing in a previously determined or random direction. (iv) Cell behaviour is predefined based on the local cytokine, oxygen, and mineral concentrations. OCL resorb both mineral and osteoid simultaneously whereas OBL only synthesize osteoid. Although the skeletal cells (OBL, OCY and lining cells) secrete OPG and RANKL, OCY synthesize significantly more than the two other cell types combined. The strain threshold for osteoid formation (FOR) is the same for the SED and EFF signals. The oxygen threshold is defined as the presence of sufficient oxygen, i.e., more than is consumed per iteration.

The *in vivo* datasets consisted of time-lapsed *in vivo* micro-CT (vivaCT 40, Scanco Medical AG, Brüttisellen, Switzerland) scans of femoral osteotomies in female, 20-week-old C57Bl/6J mice from a prior study (Wehrle et al., 2019). Briefly, osteotomies were created with a 0.66 mm Gigli wire resulting in a mean osteotomy width of 0.85 ± 0.09 mm. The femur defect was stabilised with a polyether ether ketone external fixator (MouseExFix, RISystem AG, Davos, Switzerland) with an *ex vivo* apparent construct stiffness of 23.8 N/mm (Tourolle né Betts et al., 2020) across the defect gap (Figure 1A). The *in vivo* micro-CT images were acquired every week for 5 weeks at an isotropic voxel resolution of 10.5 μm. The repeated anaesthesia, handling and radiation associated with the scans did not have a measurable impact on callus formation and remodelling (Wehrle et al., 2019). The calibration-validation split was assigned randomly *a priori* to fit the micro-MPA model’s output to the calibration group (n = 4) and provide an unbiased evaluation of the calibrated model using the validation group (n = 5). The dataset presented a large variation in gap widths spanning each osteotomy for each sample and their assignment to the group is shown in Supplementary Figure S1. This study design was essential to tune and adjust the parameters of the model, while also ensuring that the model remained generalisable, i.e., could be readily applied to different samples and expect a similar outcome.

### 2.2 Micro-multiphysics agent-based model

The micro-MPA model is based on an earlier version (Tourolle, 2019) and was expanded in this study to investigate the contributions of revascularization and remodelling to bone regeneration. The overview of the current model is depicted in Figure 1B. Briefly, the micro-MPA model coupled micro-finite element (micro-FE), micro-finite difference, and agent-based modelling to simulate revascularisation, bone formation, resorption, and tissue differentiation in a mouse femoral osteotomy defect. The micro-FE and micro-finite difference sub-models of the micro-MPA model run on open-source libraries (Boaretti et al., 2023) which are widely adopted and maintained by the high-performance computing community. The model was highly repeatable as all random seeds were fixed, i.e., two identical models running on different cores produced the same outcomes.

#### 2.2.1 Micro-finite element model

The micro-FE model was generated as a hexahedral (voxel) mesh from the input image. For each iteration of the micro-FE step within the micro-MPA model, each element’s stiffness was updated based on the mineral density (see Figure 1A). The conversion of mineral density to Young’s modulus was achieved via linear interpolation where 720.0 mg HA/cm^3^ and 395.0 mg HA/cm^3^ corresponded with 14.0 GPa and 4.0 GPa, respectively (Shefelbine et al., 2005; Mulder et al., 2007). Elements with a mineral density below 395.0 mg HA/cm^3^ were considered soft tissue and were given a value of 3.0 MPa (Simon et al., 2011; Tourolle né Betts et al., 2020). Both soft and mineralised tissue were modelled as linear elastic materials with a Poisson’s ratio of 0.3 (Claes and Heigele, 1999; Flaig and Arbenz, 2011). The stiffness of the osteotomy was determined by applying a simple uniaxial compression of 1% displacement to the cortical bone and the marrow cavity. The computed value of compressive apparent stiffness was then used to derive the loading boundary conditions, assuming combined bending and compression from ambulatory loading with an external fixator (Tourolle né Betts et al., 2020). The computed strains were then linearly scaled so that the resulting force was 10.5 N which represented the peak force determined via habitual loading (Paul, 2020). The micro-FE models were solved across 2 nodes on Piz Daint, a Cray XC30/40 system at the Swiss National Supercomputing Centre (CSCS), using ParOSol, a parallel solver optimised for micro-CT images (Flaig and Arbenz, 2011). The mechanical stimulus used for the cells within the soft tissue was effective strain (EFF), calculated as described by Pistoia et al. (2002), whereas the mechanical stimulus for the osteocytes within the mineralised tissue was strain-energy density (SED) (Schulte et al., 2013a). Both mechanical signals were Gaussian-filtered (sigma = 1.0, support = 0.8) to mitigate partial volume effects following the standard approach when evaluating micro-CT images (Bouxsein et al., 2010; Ohs et al., 2020). This signal regularisation was also previously applied to micro-FE analysis on *in vivo* micro-CT images (Schulte et al., 2013b; Tourolle né Betts et al., 2020; Paul et al., 2022). The presence of these two mechanical signals allowed tissue-independent standardisation of the various strain-related thresholds concerning the cell behaviours (Kendall et al., 2022a). This was especially relevant for this study where the osteotomy gap developed into a hard callus which presented a porous surface, resembling a trabecular structure. The mechanical signal predicted by SED alone within the soft tissue niches of these porous structures was too low to induce osteoid production (see Supplementary Figure S2). Having taken this into consideration, we used EFF to predict the mechanical stimulus for the soft tissue (Pistoia et al., 2002), assuming small strains and that the Young’s modulus of mineralised tissue was orders of magnitude greater than that of soft tissue. The osteogenic (SED_FOR_ > 0.008 MPa or EFF_FOR_ > 0.008) and osteolytic (SED_RES_ < 0.015 MPa or EFF_RES_ < 0.015) strain thresholds (Figure 1) were used globally, for all cells either occupying mineralised or soft tissue.

#### 2.2.2 Micro-finite difference model

The cytokine and oxygen concentrations were simulated using a linear backward time-centred space (BTCS) finite-difference solver. The details of the solver are described in more detail by Tourolle (2019) and Boaretti et al. (2023). The reaction-diffusion-decay (RDD) step of the micro-finite difference model consisted of many sub-steps, which were scaled with the global update interval. The stability of the RDD step was ensured for each chemical species and verified prior to the start of the study (Boaretti et al., 2023). The simulation domain was discretised at the same resolution as the micro-FE model where each element (voxel) was assigned isotropic diffusivity for each cytokine. Oxygen was assumed to have an equal diffusivity constant for soft tissue and bone. For all cytokines, we assumed mineralised tissue to be less diffusive by two orders of magnitude than non-mineralised tissue. The diffusion constant was determined to be the same for all cytokines in soft tissue since all proteins were soluble (Yu et al., 2009) and not membrane-bound (Supplementary Table S1). The distal and proximal concentrations were constant at the domain boundaries (Dirichlet) with the other boundaries being set to reflective (Geris et al., 2008). Further, cytokine binding was modelled in a reaction step that captured receptor-ligand kinetics and ligand-ligand, e.g., osteoprotegerin (OPG) and receptor activator of nuclear factor kB ligand (RANKL), interactions. The linear reaction kinetics and implementation thereof were described previously (Tourolle, 2019; Boaretti et al., 2023). The finite-difference model also included cytokine decay where each cytokine was assigned an exponential decay constant (*see* Supplementary Table S1). With every RDD step, the isotropic diffusivity for each element and chemical species was updated based on the mineral concentration at the respective element. The mineral concentration was determined by the mineralisation rate of the local osteoid secreted by osteoblasts, resulting in a time-delay which governs the dynamics of matrix mineralisation (Supplementary Material).

#### 2.2.3 Agent-based model

The agent-based sub-model of the micro-MPA model was implemented in C++. The biological cells were represented as independent, discrete agents with pre-defined behaviours (proliferation, differentiation, apoptosis, migration, protein, and extracellular matrix synthesis) based on conditions in their surrounding environment. The discretisation of the ABM was equal to the micro-FE model, i.e., each 10.5 μm isotropic voxel could be occupied by a single cell only. Voxels were considered as soft tissue if their mineral content was below 395 mg HA/cm^3^ (Tourolle né Betts et al., 2020) and could be occupied by skeletal stem and progenitor cells (SSPC), hematopoietic stem cells (HSC), vascular endothelial cells, immune cells, osteoclasts (OCL), osteoblasts (OBL) and bone lining cells. The ABM assumed that all cells were of the same size except for active OCL which were multi-nucleated clusters consisting of 3 or more OCL (Tourolle, 2019). Mineralised voxels (>395 mg HA/cm^3^) could be occupied by only pre-osteocytes or osteocytes (OCY). Since the *in vivo* conditions of the osteotomies were classified as gap healing (Shapiro, 2008), also referred to as direct transformational bone repair, the micro-MPA model did not include chondrocytes and thus fibrocartilage formation. The differentiation of SSPC along the osteogenic lineage was only permitted *in silico* if the SSPC were saturated with oxygen and within a preferable mechanical environment (SED_FOR_ > 0.008 MPa or EFF_FOR_ > 0.008) (Li et al., 2019). Cell migration for OBL and OCL is illustrated in Supplementary Figure S3 whereas all other cell types, except (pre-)osteocytes, migrated randomly. Cell migration was possible only in soft tissue and if a voxel was already occupied, the migrating cell would swap positions with the resident cell. Only OBL were not able to swap positions with OCL, as this would result in a continuous loop of formation and resorption. The parameters pertaining to migration behaviour can be found in Supplementary Table S1. Based on the discretisation of the model, the update interval for the agent-based model (dt_cells_ = 20 min) was calibrated to achieve the mean target speed of 31.5 μm/h for cell migration (Appeddu and Shur, 1994).

All cells either expressed cytokines, or synthesised extracellular matrix, or both as stated in Supplementary Table S1. The cytokines vascular endothelial growth factor (VEGF), OPG, RANKL and sclerostin (Scl) diffused, decayed, and reacted within the extracellular volume. Additionally, transforming growth factor beta 1 (TGF-beta) was either synthesised by immune cells and/or stored within the mineralised tissue to be released upon bone resorption by OCL (Wu et al., 2016). Also, all cells had cytokine receptors (RANK, VEGFR, TGFBR1, LRP5/6) which allowed the binding of free cytokines (RANKL, VEGF, TGF-beta, Scl) via reaction kinetics computed as part of the micro-finite difference model of the RDD steps. All signalling was modelled with receptor-ligand kinetics, with the respective receptors located on each cell’s surface. Furthermore, a free ligand could also bind to a corresponding free ligand, e.g., OPG could bind with RANKL to form RANKL-OPG. These pathways are visualised in Figure 1B.

The population of each cell type was dependent on proliferation, apoptosis, and differentiation. SSPC, HSC, OCL, OBL and immune cells spontaneously underwent apoptosis during any cell behaviour timestep. Only SSPC and HSC proliferated in the presence of sufficient oxygen tension and a favourable mechanical environment. Proliferation and apoptosis rates are given in Supplementary Table S2. In general, cell differentiation was possible if the precursor cell was sufficiently oxygenated with the cell’s fate depending on the mechanical environment depicted in Figure 1B (Morgan et al., 2010) or receptor binding. For example, the mechanical environment directly dictated the differentiation of OBL from SSPC (EFF_FOR_) or the inhibition of lining cells being formed from mineral lining SSPC/OBL if the EFF_FOR_ threshold was surpassed. Whereas the molecular signalling pathways especially affected the differentiation and migration of OCL including their respective pre-cursors (Nelson et al., 2012; Warren et al., 2015): The immune cells, which represented macrophages of the M2 phenotype, were able to differentiate to OCL even in a hypoxic environment (Ono and Nakashima, 2018) if 50% of RANK binding sites were occupied. Similarly, differentiation of HSC to OCL was driven by the presence of occupied RANK receptors (>50%). The differentiation of lining cells to OBL (Matic et al., 2016) was regulated, i.e., inhibited, by the presence of Scl (Ominsky et al., 2015). The differentiation of OBL to OCY was further regulated by the surrounding mineral concentration and an embedding rate which was calibrated to achieve an osteocyte density of 44′800 cells/mm^3^ (Mader et al., 2013).

Osteocytes acted as mechanosensors and synthesised cytokines that stimulate the catabolic (RANKL), anti-anabolic (Scl) or anti-catabolic (OPG) pathways according to the local SED (Klein-Nulend et al., 2013) whereas OBL and bone lining cells secreted OPG and RANKL based only on the local effective strain (Pistoia et al., 2002). The cytokine production was modelled as a linear function of the perceived mechanical signal with the parameters listed in a previous implementation (Tourolle, 2019) and the production values listed in Supplementary Table S1. Thus, VEGF and TGF-beta were independent of the mechanical environment. The expression of VEGF was modelled as a function of the cellular oxygen fulfilment which is shown in detail for each cell type in Supplementary Figure S4. OCL were the only cell type to resorb mineral and osteoid. Furthermore, OCL could only resorb as a multi-nucleated cluster consisting of 3 or more OCL (Tourolle, 2019; Boaretti et al., 2023). Resorption occurred only if more than 50% of RANK binding sites were occupied whereas osteoid production by OBL occurred only above EFF_FOR_. The directionality of osteoid synthesis and mineral resorption was defined by a polarisation function which biased the deposition of osteoid in favour of voxels with relatively high effective strain, whereas mineral resorption was biased by minimal gradients of strain energy density (Van Oers et al., 2008; Kendall et al., 2022a). Osteoid was also deposited in the same voxel the OBL occupied whereas voxels occupied by vasculature was void of osteoid. The polarisation function was previously described by Tourolle (2019). Behaviour of OBL and OCL are depicted in Supplementary Figures S3B, C.

A final set of agents, vascular endothelial cells, represented the vasculature. Vessel sprouting, extension, branching and anastomosis depended on the local concentration of VEGF, which was secreted by SSPCs, OBL, OCL, pre-osteocytes, bone lining cells, and immune cells. The VEGF-dependent growth of the vasculature was similar to an algorithm described by Checa and Prendergast (2009), in that vessel extension and sprouting may follow the VEGF gradient, a random direction or a previous direction; however, in our adaptation, vessel growth could also occur via bifurcation at pre-existing vessels (Carlier et al., 2012; Kusumbe et al., 2014). This implementation of revascularisation was previously validated where the vascular volume (Supplementary Figure S5) agreed with data obtained via micro-CT angiography at 0, 3, and 14 days post-operation in a 1.5 mm defect (Kendall et al., 2022b). Vascular growth was not mechanosensitive, and the parameters were tuned such that the oxygen tension at week 5 agreed with experimental measurements of oxygen tension in the periosteal volume adjacent to the fracture gap (Epari et al., 2008). Although the oxygen tension data was derived from an ovine study, we assumed that the physiological response between ovine and mouse models was similar (Brighton and Krebs, 1972).

#### 2.2.4 Model initialisation

The raw micro-CT scans were automatically pre-processed as described in a previous study (Wehrle et al., 2021) where the image at post-operative day 0 (POD 0) served as the initial geometry for the micro-MPA model. Initial cell seeding was defined according to Supplementary Table S3 where a uniform random distribution within all free positions in physiologically relevant regions was assumed for each cell type. SSPC and lining cells were predominantly placed on the periosteal surface and within the periosteum which was modelled as a 52.5-μm-thick layer (Chartier et al., 2018). The haematoma within the osteotomy gap was also initially occupied by SSPC albeit at a lower density. The haematoma was modelled by dilating the defect centre mask by 210 μm. Immune cells were also randomly seeded within the haematoma whereas the HSC occupied the endosteal cavity. Vascular endothelial tip cells were initialised within the periosteum and endosteum at both the proximal and distal ends. The number of tip cells was kept constant (n = 200) across all simulations and samples. Osteocytes were randomly seeded within the mineralised tissue. OBL and OCL were not present at POD 0. VEGF and oxygen concentrations were set to be 5 times greater within the initial haematoma than within the surrounding tissue (Epari et al., 2008). The surrounding tissue was assumed to be normoxic at 0.065 mol_O2_/m^3^ (Collins et al., 2015). To mitigate any initialisation effects resulting in simulation artifacts, the RDD was run for 48 iterations (1 day) prior to cell seeding. All cell receptors were then initialised as unbound. The summary of the initial conditions is defined in Supplementary Tables S2, S3. Importantly, all random seeds were fixed such that the initialisation step was repeatable.

#### 2.2.5 Model parameters

In Supplementary Table S1 we report the parameters that define the micro-MPA model and its initial conditions. These parameters capture the probability of random movement of the cells, their binding site numbers, oxygen consumption, individual cytokine production and binding sites, etc. Relevant literature was searched to identify parameter values. In lieu of missing literature values, assumptions for variables of indeterminate functions and/or values from calibration simulations were made. These assumptions were optimised via grid-search in cases when multiple parameters were dependent, and a parameter sweep when the parameters were independent. The optimal parameters were evaluated using root mean square error (RMSE) of the differences between *in silico* and *in vivo* calibration datasets. This model calibration was performed by identifying optimal parameters by minimising RMSE (Supplementary Table S1): The error was calculated independently for each volume of interest across all time points at every tissue mineral density threshold. Important examples would include bone formation and resorption which were not only determined by osteoid production and mineral resorption rates, respectively, but also OBL and OCL population size and migration. The mineralisation rate of 30 mg HA/cm^3^/day was determined by fitting the changes in between the multidensity thresholds between time points.

### 2.3 *In silico* study of osteotomy gap sizes

The original OG was randomly selected from the calibration dataset (mouse 5) and had a median gap size of 0.86 mm (minimum = 0.40 mm and maximum = 0.98 mm) along the longitudinal axis. By segmenting the distal cortical fragment, its position could be manipulated relative to the proximal fragment thereby changing the width of the osteotomy gap *in silico*. The distal cortical fragment was moved in steps of 0.15 mm, resulting in 11 models where the median OG sizes ranged from 0.41 mm to 1.91 mm. The smallest OG was limited by defect geometry whereas the largest OG was already limited by computational memory. Thus, a workaround for 1.91 mm OG was found by cropping the image ends, thus resulting in the same overall volume of the 1.76 mm model. The cell densities were identical for all models at initialisation. The setup and course of the regeneration were identical to the previous simulations.

### 2.4 Postprocessing analysis: bone morphometry and visualisation

Bone volume (BV) was calculated by counting the number of voxels above a tissue mineral density threshold and multiplying it with the voxel volume. For the analysis leveraging a multidensity threshold approach, thresholds started at 395 mg HA/cm^3^ and increased by steps of 25 mg HA/cm^3^ until the maximum threshold of 720 mg HA/cm^3^ (Tourolle né Betts et al., 2020). The maximum threshold represents the density threshold for cortical bone (Bouxsein et al., 2010). The binary thresholds were computed at the lowest mineral threshold of 395 mg HA/cm^3^ (Tourolle né Betts et al., 2020; Wehrle et al., 2021), which represented initial woven bone. To measure the degree of mineralisation and its progression, a higher mineral threshold of 645 mg HA/cm^3^ was applied and the ratio of lowly mineralised to highly mineralised tissue (BV645/BV395) was determined (Tourolle né Betts et al., 2020). Volumes of formation, quiescence, and resorption (FQR) were calculated by the difference between two thresholded images of consecutive time points to establish the respective bone formation rate (BFR) and bone resorption rate (BRR) (Paul et al., 2022). Importantly, the postprocessing analysis pipeline was equal for both *in vivo* and *in silico* samples.

Cortical bone morphometry was expressed using medullary area (Ma.Ar), cortical bone area (Ct.Ar), cortical thickness (Ct.Th), and intracortical porosity (Ct.Po). These values were computed for the samples of the validation group at POD 35 following the guidelines for the assessment of bone microstructure using micro-CT (Bouxsein et al., 2010). For the evaluation of bone regeneration, four volumes of interest were created automatically based on the approach outlined by Tourolle né Betts et al. (2020) from the post-operative measurement: initially void of bone, the defect centre (DC) and defect periphery (DP) spanned the osteotomy gap (OG), containing the endosteal and periosteal callus. The fragment centre (FC) initially contained both medullary cavities and cortices, whereas the fragment periphery (FP) tracked the bone formation periosteal to the old cortices. For the quantification of BV/TV, the bone volumes within each volume of interest were normalised to the respective central volume of interest, i.e., DC/DC, DP/DC, FC/FC, FP/FC. OG widths were calculated via tracing rays starting at the femur’s longitudinal axis in a perpendicular direction. The number of rays which fit entirely between the cortices were then multiplied with the voxel height of 10.5 μm, resulting in an OG width for each direction (*see* Supplementary Figure S1). The median distance was reported from the sampled distribution of all rays across the gap. Vascular volume in each volume of interest was measured by counting the number of connected vessel cells and multiplying with the corresponding voxel volume. The renderings of the three-dimensional data were obtained using ParaView (Kitware, Version 5.10; Clifton Park, NY, United States).

### 2.5 Model evaluation and statistical analysis

By comparing the micro-MPA model of bone regeneration with time-lapsed *in vivo* micro-CT data, the similarities and differences in healing patterns could be identified and quantitatively evaluated. To validate the model, we reported the goodness of fit of the micro-MPA model’s predictions with the *in vivo* data using RMSE. The same statistical evaluation was applied from a previous study between two *in vivo* groups (Wehrle et al., 2019): repeated measurements two-way ANOVA with Geisser-Greenhouse correction and Bonferroni correction was performed in Python (statsmodels, 0.13.5) between the *in vivo* and *in silico* groups. The comparison of morphological parameters between groups was performed with one-way ANOVA analysis followed by Tukey’s multiple comparisons test. Values are given in mean ± standard deviation unless indicated otherwise. *p*-values smaller than 0.05 were considered significant.

## 3 Results

All *in silico* samples of the calibration and validation groups showed bridging and healing of the OG. The simulations of 5 weeks of healing took up to 12h, with 12 Mio elements and 776 thousand cells, on average. A single cell and reaction-diffusion-decay step took up to 20 s, whereas solving a single micro-FE analysis took around a minute on average. The *in silico* micro-MPA model simulated the presence of bone, immune and vascular cells and their interaction with the local environment, the interaction between the cells at the same isotropic voxel resolution of the micro-CT image. In the sections below, the progression of bone regeneration observed *in vivo* and *in silico* for a representative sample (mouse 7) is presented from the calibration group. Then, the outputs of the *in silico* model are reported and quantitatively compared to the *in vivo* measurements for the calibration and validation datasets. This comparison yielded the model’s accuracy in simulating bone regeneration based on the bone volume fraction. Finally, the model’s sensitivity to osteotomy gap sizes is shown and the hypothesis regarding non-union evaluated.

### 3.1 Progression of bone regeneration: representative sample

**FIGURE 2:**
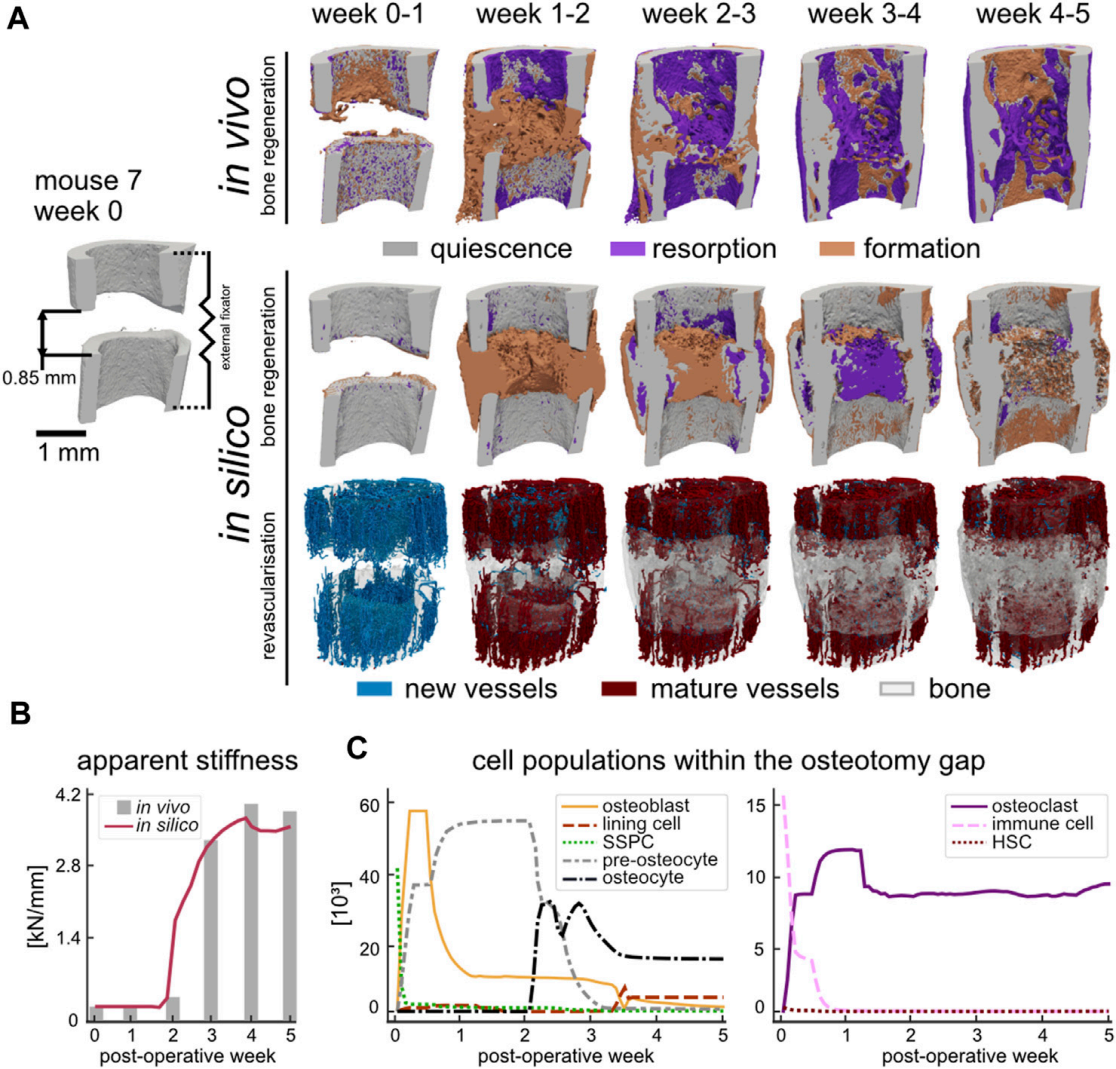
**(A)** Qualitative comparison of bone regeneration using representative time-lapse images (threshold: 720 mg HA/cm^3^) of the osteotomy gap of the same animal *in vivo* and *in silico*. Visualisation of bone quiescence (grey), formation (orange), and resorption (purple) was obtained via registration of micro-CT data and Boolean operations. New vessels (blue) formed as a result of revascularisation which then connected to other vessels and matured to red vessels at the later time point. The bone volume of the later timepoint (quiescence and formation) was rendered opaque to provide a visual reference. **(B)** Apparent stiffness of the regenerating simulated (red) and *in vivo* (grey) femur measured via micro-FE. **(C)** Cell populations during simulated bone regeneration which include the skeletal cell lineage (left) and the hematopoietic cell lineage (right).

As shown in Figure 2A, the course of bone regeneration for the *in vivo* osteotomy defect model was mainly characterised by early formation of osteoid which subsequently mineralised, bridging the defect gap. Bone resorption was active as of the first week (week one to two, BRR_FC_ 0.45%/day) and became more prominent as the bone regenerated, significantly increasing to peak resorption between week 2–3 (BRR_FC_ 0.62%/day, *p* < 0.05). Bone formation coupled with resorption resulted by week 5 in a regenerated structure which was mechanically optimised: a 11.1% decrease in bone volume fraction (BV_395_/TV_OG_) from maximum only resulted in a 3% decrease from maximum apparent stiffness (Figure 2B) for mouse 7. The *in silico* model also demonstrated a similar progression where the defect gap is bridged by week 2 and the final apparent stiffness at week 5 only marginally decreased (4.6%) to 3.50 kN/mm from the maximum at week 4. Whereas the simulated bone volume fraction (BV_395_/TV_OG_) at week 5 decreased by 12.4% from its maximum at week 4. Qualitatively, the simulated structures shared several common features with the *in vivo* data: namely, larger bone volume in regions further from the external fixator, the formation of pores within the regenerated cortex, the reduction of stress concentrations and intramedullary trabecular structures. Interestingly, the *in silico* micro-MPA model was able to simulate bone remodelling between weeks 4 and 5 (Figure 2A) as a result of emergent behaviour where OCL assembled to clusters (Supplementary Material Supplementary Figure S6) to resorb the bone in response to high RANKL (Figure 5). In contrast to phenomenological models where the clustering behaviour is strictly enforced on the tissue level (Van Oers et al., 2008), this was observed in the micro-MPA model as a result of a large collection of cells with simple rules. Since the magnitude of external mechanical loading was assumed constant during the regeneration period and no new stimulus was introduced, the *in silico* model approached a steady state as it had adapted to the mechanical stimulus by week 5. The initial haematoma within the osteotomy gap was populated with immune cells and SSPC (Figure 2C) which represented the inflammatory phase of bone regeneration. Although the haematoma was modelled as hyperoxic, the large presence of stem cells rendered the environment hypoxic after POD 1, triggering a spike in hypoxia-induced VEGF synthesis, followed shortly by vascular infiltration (Figure 2A, week 0–1). The production of TGF-beta by the immune cells and the presence of sufficient oxygen close to the cortices permitted the SSPC to differentiate to OBL (Figure 2C, week 0–1) which in turn started synthesising osteoid. By POD 2, the OBL population had peaked at 57′040 cells for mouse 7 across the OG. This differentiation along the osteogenic lineage resulted in a steep loss of SSPC across the OG, however, a large concentration of SSPC remained in the periosteum (65% by POD 3). As vessels began to cross the OG, the osteoid producing cells shortly followed. By day 3, OBL constituted just over 50% of the cell population within the OG. With a growing callus, the proliferating OBL population either differentiated to pre-osteocytes and were embedded within the mineralising osteoid or underwent apoptosis (Figure 2C, week 0–3). As of post-operative week 3, the osteocyte population outgrew the pre-osteocyte population and represented 58.9% of the total cell population. The OBL trans-differentiated (Figure 2C, week 3–4) to lining cells which retained the ability to re-differentiate to OBL given sufficient mechanical stimulus (Figure 2C, week 3–4). At the final timepoint there were 3 lining cells for each OBL (6.2%), however OCY were still by far the most prevalent cell type (52%) across the OG.

Meanwhile, either the immune cells fused, or HSC differentiated directly to form OCL (Figure 2C, week 0–1) which lined the non-loaded fragments and slowly began resorbing the mineralised tissue due to high RANKL concentration. However, the contribution of the HSC remained limited to the population of OCL in the osteotomy gap since they were seeded within the FC region. The OCL resorbed volumes high in RANKL, which until the fracture gap was bridged, would be mainly around the cortices due to the absence of mechanical stimulation (Figure 2A, week 0–2). Once the synthesised osteoid began to mineralise, trabecular structures formed which then gradually increased in thickness until the osteotomy gap was fully bridged. Due to the time-delay in mineralisation, an overproduction of osteoid resulted in a large hard callus (Figure 2A, week 1–2). The embedded (pre-)osteocytes started RANKL synthesis due to minimal mechanical loading, and the mineralised tissue was gradually removed until the structure was of similar outer diameter to the pre-existing cortex (Figure 2A, week 2–5).

### 3.2 Model validation via comparison to *in vivo* data

**FIGURE 3:**
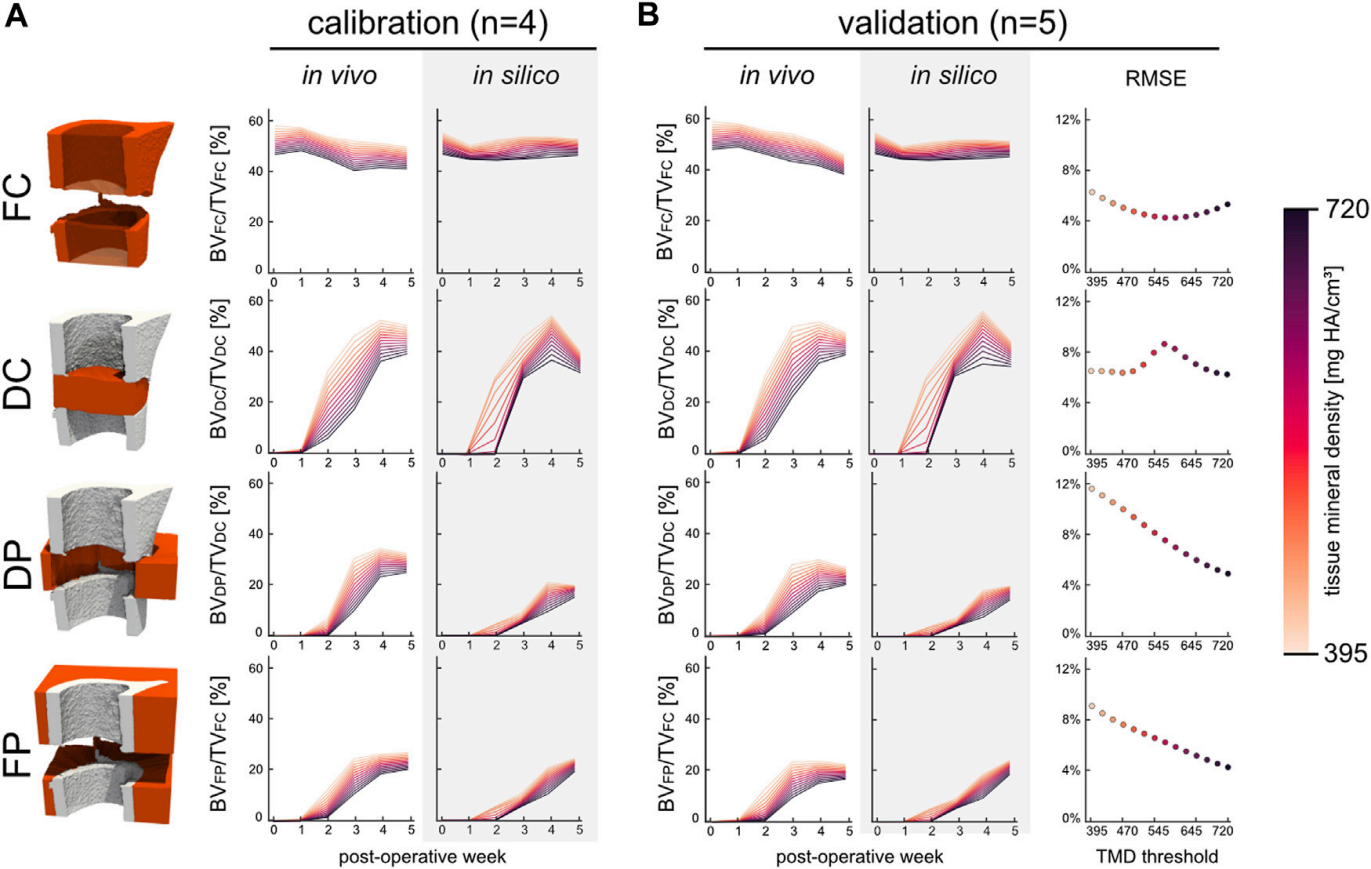
The development and kinetics of bone formation and mineralisation in the four volumes of interest for the calibration (n = 4) and validation (n = 5) groups. **(A)** Time course of bone volume fraction and mineralisation which are both threshold dependent. **(B)** Bone volume fraction for the validation group which includes the RMSE scoring with respect to the *in vivo* data. The error was evaluated across all time points at each threshold for all four volumes.

The micro-MPA model’s parameters and cell rules were determined by fitting the model’s output during the development stage to a calibration dataset (Supplementary Table S1). The results of this process are shown in Figure 3A. Importantly, there was no statistically significant difference in bone volume fraction (720 mg HA/cm^3^, *p* = 0.82) between the *in vivo* calibration and validation datasets (Figure 3B). As part of the calibration efforts, we investigated the effect of varying the random seeds and found that the model was not sensitive to random perturbations in the initial distributions. The validation dataset (n = 5) was chosen *a priori* with the aim to determine the model’s ability to fit to the *in vivo* bone volume during bone regeneration. The RMSE was calculated for each tissue mineral density (TMD) threshold within the specified range of 395 and 720 mg HA/cm^3^ in increases of 25 mg HA/cm^3^ and averaged across the 6 time points. The minimal error in the FC volume was achieved at TMD threshold of 595 mg HA/cm^3^. However, this threshold resulted in a near maximum RMSE of 8.2% within the DC volume (max RMSE of 8.5% at 570 mg HA/cm^3^). Both the DP and FP volumes presented similar characteristics regarding the RMSE which was inversely correlated (Pearson correlation coefficient of −0.997 and −0.996, respectively) with the TMD threshold. Thus, the minimum cost was achieved for the volume spanning the OG by selecting the highest TMD threshold 720 mg HA/cm^3^ resulting in an overall RMSE of 5.5%.

**FIGURE 4:**
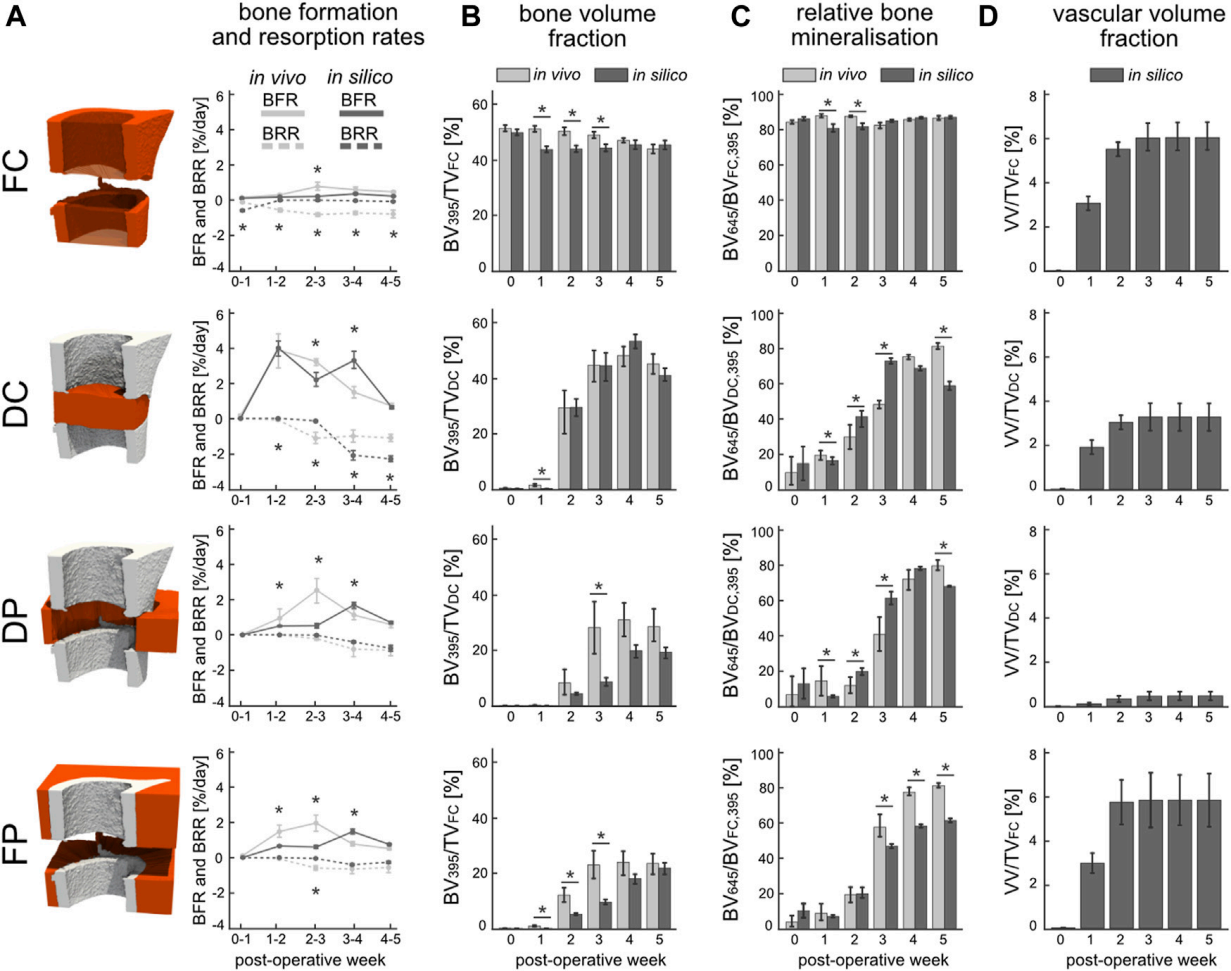
Evaluation of *in silico* (dark) and *in vivo* (light) bone regeneration via micro-CT parameters in the four volumes of interest: fragment centre (FC), defect centre (DC), defect periphery (DP), and fragment periphery (FP). **(A)** Bone formation rate (solid line) and bone resorption rate (dashed line) reported in percent per day. **(B)** Bone volume (BV) normalised to the centre TV, i.e., DC for DC and DP, FC for FC and FP. **(C)** Degree of bone mineralisation reported as a ratio of highly mineralised bone (≥645 mg HA/cm^3^) to the total mineralised volume (≥395 mg HA/cm^3^). **(D)** Vascular volume (VV) normalised to the centre TV. *indicates *p*-value <0.05 determined by repeated measurements two-way ANOVA with Geisser-Greenhouse correction and Bonferroni correction. N = 9 for both groups.

From week 1 to week 2 a significant increase in bone formation was detected (Figure 4A) in the total VOI (TOT = DC + DP + FC + FP) *in silico* (*p* < 0.0001) and *in vivo* (*p* < 0.0001), as a result of the successful transition from the inflammatory to the regenerative phase. However, the progression from the repair to the remodelling phase as indicated by the BRR occurred *in vivo* at week two to three in the DC region and at week three to four in the DP region. Similar trends were obtained *in silico* for the BFR and BRR within the DC and DP volumes, albeit the progression to the remodelling phase occurred at week three to four in the DC region. Lower BFR was calculated from the *in silico* samples for the initial 3 weeks than was for the *in vivo* samples in both DP and FP volumes whereas the BRR closely tracked each other. BFR and BRR followed different trends in the FC volume which resulted from significant differences between the bone volume fractions of the *in silico* and *in vivo* samples (*p* < 0.05) as of week 1 until week 3 (Figure 4B). Similarly, bone volume fraction in FP was significantly higher (*p* < 0.05) from week 1 to week 3 for the *in vivo* samples. Importantly, when considering the OG volume (DC + DP) no significant difference in bone volume fraction could be observed between the *in silico* and *in vivo* samples (*p* = 0.68). The mineralised bone fraction or relative bone mineralisation (BV_645_/BV_395_) (Figure 4C) indicates the maturity of the mineralised tissue as it progresses from osteoid to compact bone. No significant differences were observed between the two groups at POD 0. Early resorption of the FC *in silico* resulted in a significantly lower bone mineralisation up until week 2 when compared to the *in vivo* group. Both the DC and DP volumes reported similar bone mineralisation trends for both groups which thus also resulted in significant differences between groups at weeks 1, 2, 3 and 5. The mineralisation modelled by the micro-MPA appears more dynamic whereas the *in vivo* data suggest a more bounded behaviour that targets a mineralised bone volume fraction of 85%. The mineralised bone volume fraction was significantly lower in the *in silico* group at week 5 than in the *in vivo* group. The vascular volume fraction (VV/TV) (Figure 4D) reports the growth and extent of the vascular network and includes both vascular endothelial tip and stalk cells. Revascularisation precedes bone formation in the DC and FP regions by a week and reaches a maximum average volume fraction of 3.7% and 5.9%, respectively, at week 3. Little vasculature is present within the DP volume due to an overall low VEGF concentration which results from a low cell population resulting in a VV/TV of 0.6%. Vascular growth had little effect on the bone formation and resorption in the FC volume, however, had a large effect on initial bone formation in the DC and DP volumes by providing the necessary oxygen for stem cell differentiation in the *in silico* model.

When comparing the morphology (Table 1) of the regenerated structures at POD 35, the *in silico* model reported a significantly smaller mean cortical area (Ct.Ar) than the *in vivo* group. Due to the relatively large variance in the datasets, the mean medullary area (Ma.Ar) was not significantly different. Interestingly, the cortical thickness (Ct.Th) was significantly greater in the *in vivo* dataset than the simulations. Given the nearly identical intracortical porosity (Ct.Po), this indicated that periosteal and endosteal surfaces were structured with voids which also effectively reduced the mean cortical thickness.

**TABLE 1:** Morphological parameters evaluated across the original defect gap region at POD 35 for the validation group. The parameters are calculated as described by Bouxsein et al. (2010). Values are given in mean ± standard deviation. *p*-values were computed between the *in vivo* and *in silico* groups where a value smaller than 0.05 was considered significant.

Parameter	*IN VIVO*	*IN SILICO*	*p*-value
MEDULLARY AREA (MA.AR)	0.61 ± 0.31 mm^2^	0.42 ± 0.22 mm^2^	0.442
CORTICAL AREA (CT.AR)	0.92 ± 0.10 mm^2^	1.33 ± 0.15 mm^2^	0.016
CORTICAL THICKNESS (CT.TH)	0.25 ± 0.01 mm^2^	0.21 ± 0.01 mm^2^	0.028
INTRACORTICAL POROSITY (CT.PO)	9.03% ± 4.80%	7.96% ± 5.10%	0.815

### 3.3 Mechanical stimulation of newly mineralised tissue mediates the dynamics of cytokine expression involved in bone remodelling

**FIGURE 5:**
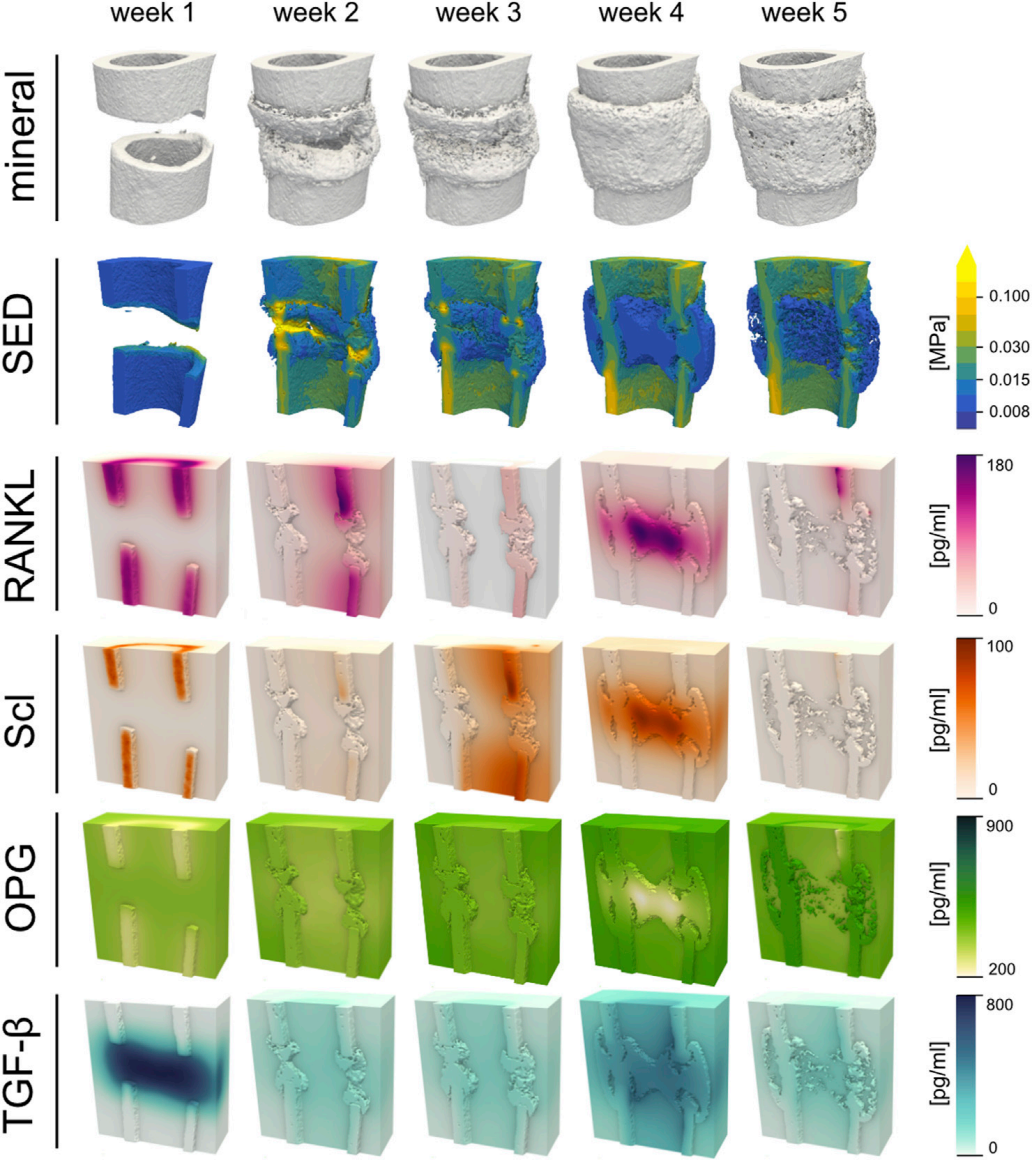
Renderings of the simulated cytokine concentrations - namely, Receptor Activator of Nuclear Factor Kappa-B Ligand (RANKL), sclerostin (Scl), Osteoprotegerin (OPG), and Transforming Growth Factor-beta (TGF-beta)—for a representative sample (mouse 7) are shown for each post-operative week. The mineral structures were thresholded at 720 mg HA/cm^3^ and for visualisation purposes, the strain-energy density (SED) i.e., the mechanical signal only within the mineralised tissue is shown in the second row. The osteogenic threshold was set to 0.008 MPa whereas the threshold for bone resorption was 0.015 MPa.

During the inflammation phase (week 1), which was characterized by high concentration of TGF-beta as a result of the large population of active immune cells, RANKL and Scl concentrations were increased within the cortical regions, while OPG was decreased, on account of the low SED in the osteotomized cortices (Figure 5). As the simulation progressed into tissue differentiation and formation (week 2), concentrations of RANKL and Scl decreased, and that of OPG increased, due to the partial bridging of the cortices that allowed strain to be transmitted across some parts of the osteotomy gap. However, the phenomenological association between SED and the production of these three cytokines changed as formation peaked and remodelling began (weeks 3 and 4): RANKL and Scl increased, and OPG decreased, because by this point in time, the osteoblasts had become embedded in the newly formed mineralized tissue and had differentiated into osteocytes which began producing RANKL. This coincided with an increase in Scl and a decrease of OPG production within the callus. Importantly, newly mineralised tissue had yet to contain embedded osteocytes and thus this tissue was not capable of secreting cytokines in response to mechanical stimulus. This introduced a time-delay in the system, which was most apparent during week 3, especially for RANKL (Figure 5). An increase in TGF-beta concentration was observed during this time due to the release of latent TGF-beta within the mineral matrix as the tissue begins to remodel. The high concentration of TGF-beta induced a small osteogenic response measured by a small increase of the osteoblast population. This population growth was primarily limited by low SSPC numbers that late into the regeneration period. Week 5 was characterised by a resorption of the hard callus and importantly, the embedding RANKL-producing osteocytes. During this late stage of healing, RANKL and Scl production was limited to local regions. Across all timepoints, RANKL was the most dominant cytokine and most closely linked with the solid tissue mechanics. RANKL is followed by Scl in potency, with OPG being the least responsive to mechanical strain.

### 3.4 Simulation of non-union

**FIGURE 6:**
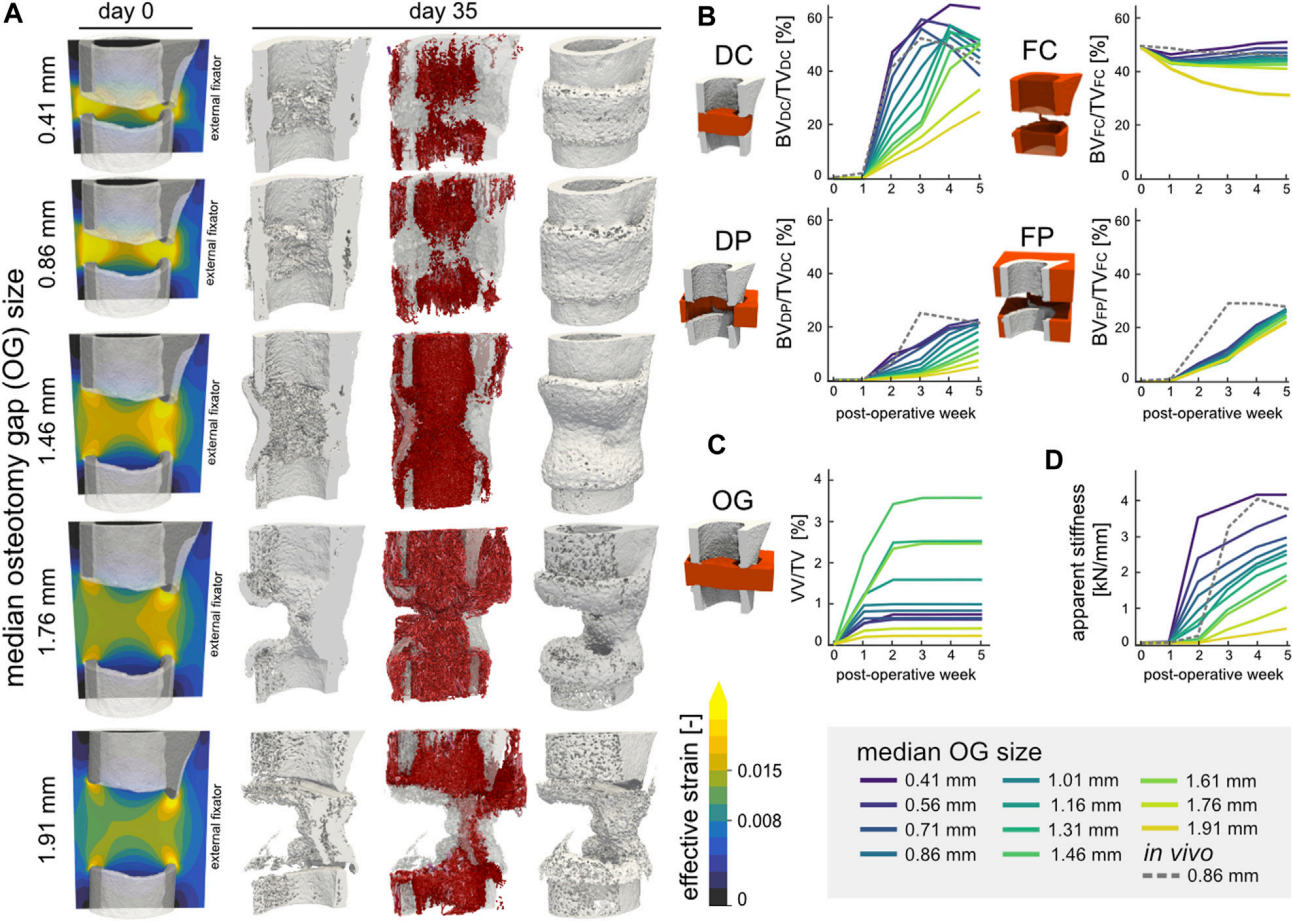
**(A)** Representative renderings of the investigation of the osteotomy gap size (n = 11) on regeneration outcomes. The initial geometries are shown for 5 configurations (median gap size 0.41 mm, 0.86 mm original, 1.46 mm, 1.76 mm, and 1.91 mm) for mouse 5. The distribution of effective strain (EFF) is shown across the cut plane in microstrain. The osteogenic threshold was set to 0.008 whereas the threshold for bone resorption was 0.015 in the soft tissue. The final structures at POD 35 are shown (cut and full image; threshold: 720 mg HA/cm^3^) including the simulated vasculature (purple-red) which is qualitatively mapped based on vessel length. **(B)** Development and kinetics of bone formation in the four volumes of interest for the 11 samples. The 0.86 mm *in vivo* sample is represented by the dashed line. **(C)** Vascular volume fraction within the osteotomy gap. **(D)** Development of the osteotomy gap’s apparent stiffness.

Varying the gap size of the defect resulted in different healing outcomes, with ultimately non-union occurring at the largest gap size of 1.91 mm. Bridging of the defect gap after 5 weeks was seen for all ten gap sizes below 1.91 mm; however, the extent of bridging (Figure 6A) and overall amount of bone formed (Figure 6B) differed. The simulations above a median gap size of 1.46 mm presented reduced bone formation of the medial cortex, arising from the variance in gap size within the defect. The DC volume presented non-linear response to the defect gap size where larger gaps resulted in greater bone density, however only up to 1.46 mm. In the FC, DP, and FP volumes, the larger the gap size the lower the bone volume fraction. Similar to the bone volume fraction, the response of the vascular volume fraction was also non-linear across the osteotomy gap (Figure 6C). The largest vascular volume fraction of 4.59% was observed in the 1.46 mm sample, whereas the 1.91 mm sample had the lowest vessel density with just 0.24%. Larger defect sizes also resulted in reduced OG stiffness of the final construct (Figure 6D). Overall, there was a high negative linear correlation of −0.971 (Pearson correlation coefficient) between the median OG size and apparent stiffness after 5 weeks.

## 4 Discussion

The proposed *in silico* micro-MPA model successfully simulated bone regeneration comparable to *in vivo* regeneration across the osteotomy gap. We demonstrated that our model could simulate the bone regeneration process from a phenomenological perspective and was validated for predictions of bone volume fraction. Importantly, the model captured the process of remodelling where the regenerated structure changed in form in response to mechanical loading, by allowing resorbed voxels to re-differentiate or mineralise *de novo,* and predicted non-union for critical gap size. As a result, the model included all key processes by covering tissue formation, differentiation, and resorption.

To date, only a handful of computational studies on bone regeneration have attempted to validate their simulation results based on the comparison of *in silico* data of individual animals to *in vivo* data. To our knowledge, none have leveraged longitudinal *in vivo* data to compare their results. This is a consequence of past studies assuming idealised geometries for their models instead of *in vivo* data (Borgiani et al., 2021). We would like to mention studies which drew comparisons to the underlying data, albeit still considering the absence quantification of error and statistical tests of their simulations: Byrne et al. (2011) plotted the apparent bending stiffness obtained from an experimental study against the simulation results. Importantly, the experimental study defined clinical healing by measuring a bending stiffness of 15 Nm/deg; however, the *in silico* results severely overestimated the rate of healing by 17 weeks. OReilly et al. (2016) instead compared the area of mineralised tissue simulated by their *in silico* model to histological data. The study by Repp et al. (2015) stands out, as they quantitatively compared simulation outcomes with reference experimental data. However, by fitting to averaged experimental data, they were unable to score their predictions against novel data, falling just short of validation. We scored the simulation output using RMSE since it is intuitive to interpret, and it enables predictive precision. However, the RMSE is sensitive to the scale of the data and is biased to favour results of smaller magnitude. The identification of a more optimal cost function remains an open task, especially since the proposed model of bone regeneration is the first to be quantitatively scored. Furthermore, the proposed micro-MPA model’s spatial discretisation ensured that spatial relationships were accurately represented at the same scale of the *in vivo* data and facilitated seamless communication between the sub-models. The presented micro-MPA model closely approximates the tissue continuum with resolution of 10.5 μm. Prior state-of-art reported a resolution of 10 μm for their agent-based models (OReilly et al., 2016; Jaber et al., 2022). Thus, the micro-scale resolution of the micro-MPA model contributes to the effort of approximating the intricate spatial dynamics involved in bone regeneration. The temporal discretisation of the sub-models, i.e., the timesteps for each iteration, also required careful consideration. The timestep governing the cell and vasculature update was determined by the migration speed and the voxel size whereas the timestep for the RDD was limited by its stability. The timestep of the micro-FE of 8 h (24 cell updates) was based on previous work and results in a finer temporal discretisation when compared to other models (Repp et al., 2015; OReilly et al., 2016; Jaber et al., 2022). Further research should consider the time interval of each micro-FE analysis iteration and consider asynchronicity to also better reflect the diurnal cycle of mice (Bains et al., 2018).

From a quantitative perspective, the model performed well in simulating bone regeneration across the osteotomy gap, as no difference was observed between the *in silico* and *in vivo* groups for the volume spanning the osteotomy gap. Regarding the pattern of the bone volume fraction across the samples in both the calibration and validation groups (Figure 4), the model simulated similar bone changes across samples. Evidently this characteristic led to amplified differences between the *in silico* and *in vivo* data in some volumes of interest, as shown in Figure 3B. The divergence of bone volume fractions within the FC region was explained by the elevated BRR for the *in silico* group. Although the fragment ends are known to resorb, especially for human patients presenting a fracture which is treated conservatively to promote endochondral ossification, we did not see any resorption in the *in vivo* model. This discrepancy is probably explained by either the mouse model itself or the quality of surgery where the vascular supply was not disrupted near the fragment ends. This finding would warrant further study to elucidate bone resorption during the inflammatory phase and identify which pathways suppress this catabolic phenomenon.

The temporal predictions of cell populations within the osteotomy gap reported in Figure 2 qualitatively agreed with the literature (Wilsman et al., 1996; Marsell and Einhorn, 2011): The inflammatory phase, identified by the presence of immune cells expressing TGF-beta in the micro-MPA model had subsided by POD 7 (Cho et al., 2002). Liu et al. (2019) reported osteoblast precursors (Osx+) within the defect centre in mouse model of bone regeneration as early as POD 3 with the peak remaining until POD 7. This peak was present in the micro-MPA model as early as POD 2, albeit lasting for 2 days. Remodelling in gap healing models was observed after bridging of the defect gap (week 3, Figure 2) which is accompanied by the resorption of woven bone segments by multi-nucleated osteoclasts (Shapiro, 2008). However, we identified a gap in the literature whereby the density and spatial distribution of various cell types, historically referred as histodynamics, during bone regeneration have not been completely investigated or are not applicable for osteotomy gap healing. OBL account for 4%–6% of total resident cells in bone whereas OCY account for up to 95% of all bone cells (Capulli et al., 2014). Our results on average show a final OBL population of 3.2% and an OCY population of 92.7% within the mineralised tissue across the osteotomy gap. The other resident cells in the simulated bone were lining cells and pre-osteocytes. Since the vasculature was not accounted for by Capulli et al. (2014) it was also not included in this analysis. Nonetheless, the predicted vascular volume of the *in silico* 1.46 mm osteotomy gap agreed qualitatively with experimental data obtained via *ex vivo* micro-CT angiography (Morgan et al., 2012; Kendall et al., 2022b) of a 1.5 mm osteotomy performed in mice. However due to the inherent size of capillaries (<7 μm) the contrast agent is not guaranteed to perfuse along the capillaries let alone be of sufficient contrast where imaging is possible (Weber et al., 2008). Furthermore, not all capillaries are perfused when the specimen is at rest (Secomb et al., 1995). These limitations in addition to biocompatibility issues makes *in vivo* time-lapsed micro-CT angiography images of the vascular network including capillaries extremely hard (Bouxsein et al., 2010; Nebuloni et al., 2013; Wälchli et al., 2021). Additionally, the micro-MPA model is only able to simulate capillaries and not larger vessels which would result in an overestimation of vessel number yet simultaneously underestimated vessel volume. This is why the vascular volume was calibrated such that the oxygen tension across the osteotomy gap also agreed with the literature (Brighton and Krebs, 1972; Epari et al., 2008). The proposed model does not include mechanosensation of the endothelial tip cells (Peiffer et al., 2011; Carlier et al., 2012) which contrasts with other revascularisation algorithms (Checa and Prendergast, 2009; OReilly et al., 2016). With the introduction of an inhibitory strain threshold for the tip cell, the micro-MPA model would have needed to include a tissue type which could be synthesised in a hypoxic environment, i.e., cartilage (Burke and Kelly, 2012; OReilly et al., 2016). Since we did not see any atrophic non-unions in the *in vivo* data (Wehrle et al., 2019), we assumed the mechanical environment was permissive to vascular growth. In addition to the addition of inhibitory strain thresholds, further research will explore the impact on vessel growth rates in response to mechanical stimuli as the vasculature has been shown to be mechanosensitive (Lu et al., 2011; Ruehle et al., 2020).

The final phase of our study delved into exploring the impact of osteotomy gap size on regeneration outcomes *in silico by* simulating a range of distraction lengths. Notably, larger osteotomy gaps were associated with inferior outcomes, as evidenced by a decline in the apparent stiffness of the regenerated structure. While this correlation aligns with the literature and corroborated by a previous study investigating osteotomy gap sizes at 0.85 mm and 1.45 mm (Tourolle né Betts et al., 2020), our approach is novel within the context of *in silico* agent-based modelling of bone regeneration. It is important to acknowledge that the presented micro-MPA model, while hinting at the influence of larger osteotomy gaps, was not validated for these scenarios. Despite this limitation, the model predicted typical experimental outcomes without altering any parameters. The adverse effects observed in larger gaps may be attributed to factors like potential shortcomings in mechanical and cytokine signalling cues. One plausible explanation is that as the osteotomy gap size increases, the distance from the centre of the gap to the cortices also grows, which raises the likelihood of cytokines decaying before reaching their intended receptors. This greater diffusion distance not only diminishes the magnitude of cytokine signalling but also introduces a temporal delay in the process. Furthermore, when we applied a force-controlled loading of the femur with a 10.5 N load along a larger osteotomy gap, it resulted in lower local mechanical strains in the soft tissue compared to the same load applied along a shorter osteotomy gap. However, when we increased the force boundary condition for the 1.91 mm osteotomy gap sample until we eventually observed a union, the local strains were classified as inhibitory to bone regeneration. This observation leads us to posit that, in the proposed micro-MPA model’s prediction of a non-union, the SSPC and lining cells near the fragment ends experienced sufficient mechanical stimulus, while the central area of the defect gap received insufficient mechanical stimulus and oxygen. An interesting avenue for future exploration could involve the inclusion of adipocytes and chondroblasts, which have been known to differentiate from SSPC in low-strain environments (Sen et al., 2008) and high-strain environments (Checa et al., 2011), respectively. In addition, by including cartilage the model could demonstrate the similarity of using SED and EFF for thresholds governing tissue differentiation, resorption, and formation. Although the thresholds presented in this study were based on research by Li et al. (2019) and are in support of the study on biophysical stimuli by Isaksson et al. (2006), they fall short of fitting into the bigger picture set by previous *in silico* models.

One of the strengths of our proposed micro-MPA model was the ability to simulate the influence of the local mechanical environment on cellular processes of bone regeneration. By using micro-FE analysis, our model was able to capture the mechanical loading of the bone during the healing process at the tissue level. This allowed the simulation of the effect of mechanical stimulus, driving the differentiation of SSPC to OBL and eventually the production of osteoid. Another key advantage of our proposed model was the ability to simulate the interactions among different cells and cytokines during the healing process. By incorporating a range of cellular and molecular processes, including cytokine binding, receptor-ligand kinetics, and ligand-ligand interactions, and simulating these processes in a dynamic environment, our model was used to examine the complex spatial and temporal dynamics of the regeneration processes. We were able to simulate physiologically relevant cellular interactions and processes such as hypoxia-induced revascularisation and resorption events by OCL clusters (Supplementary Figure S6) in response to RANKL. This strength was presented in Figure 5 where the cytokine concentrations were rendered and compared with the local mechanical environment. By varying the size of the osteotomy gap, we showed the importance of these local mechanics in driving regeneration as the increased diffusion lengths and impacted cytokine concentrations led to worse healing outcomes. In our simulations, we observed the resorption of the cortices in response to inadequate mechanical stimulation. For example, if the resorption of the cortices outpaced the cell differentiation and bone formation, the simulation resulted in structures with inadequate stiffness: the 1.76 mm sample exhibited an apparent stiffness of only 25.7% compared to the 0.86 mm *in vivo* sample. The different healing patterns illustrated how the micro-MPA model could provide insights into cellular and mechanobiological mechanisms leading to non-union.

The micro-MPA model presented limitations that need to be highlighted. Firstly, there was a discrepancy in bone mineralisation, with the *in vivo* bone mineralisation outpacing that seen *in silico*. Although the mineralisation speed was calibrated from the time-lapsed *in vivo* data of the calibration group, the model’s output was not in agreement. This discrepancy was illustrated in Figure 4 where the *in silico* model performed best at a tissue mineral density threshold of 720 mg HA/cm^3^ and the RMSE increased as the tissue mineral density threshold decreased. Furthermore, the model could have a bias to favour higher thresholds, overall favouring smaller fractions of volumes of interest. This could be addressed by including an accuracy measurement such as the Sørensen–Dice coefficient to take spatial distribution into account. It is important to acknowledge that newly mineralised tissue in the proposed model lacked embedded osteocytes and therefore could not secrete cytokines in response to mechanical stimuli, potentially limiting the model’s ability to fully represent the biological processes involved in bone regeneration. A different approach to modelling *in vivo* mineralisation should perhaps consider the effects of the local vascular supply, interstitial fluid flow, or mechanical loading which have all been shown to affect mineralisation (Liu et al., 2019; Miyamoto et al., 2021). Another limitation is a consequence of the dataset used to calibrate the micro-MPA model which, e.g., only includes histology of the final time point. Since no cartilage was observed within the histology slices (Wehrle et al., 2019), the assumption was made that the osteotomy gap did not experience excessive strains to induce a chondrogenesis and underwent direct transformational bone repair, i.e., gap healing (Shapiro, 2008). However, previous studies of bone regeneration have identified cartilage tissue even with external fixation of the femur in similar gap healing murine models (Morgan et al., 2010; Marsell and Einhorn, 2011; Borgiani et al., 2019). Thus, by not modelling cartilage, the micro-MPA model misses important mechanical cues early in the regeneration process. Also, the validation of the bone volume fraction does not capture the early inflammation dynamics nor the deposition of osteoid. Due to the longitudinal study design of the underlying dataset, validation of these important phases of bone regeneration was not possible. Intravital imaging could address this limitation by allowing the inflammatory and early callus formation to be imaged *in vivo* and used for validation similar to the bone volume fraction for this study. Due to these limitations, this model may only serve for future investigations of rigidly stabilised defect gaps which only present successful healing outcomes. Furthermore, the micro-FE analysis was based on the assumption of linear elastic material properties, which may not fully capture the precise behaviour of soft tissue or osteoid under loading. In addition, our revascularisation algorithm did not consider intra-cortical vasculature, a simplification which was compensated by increasing the oxygen diffusivity within the mineralised tissue to ensure sufficient oxygenation of the cortical bone. However, the greatest limitation of the revascularisation algorithm was the initial conditions and the absence of data to sufficiently validate the results. Although previous research had shown good agreement of the predicted vascular volume with experimental data, the model’s tip cells had been seeded using micro-CT angiography images of distraction osteotomies (Kendall et al., 2022b). Thus, the inclusion of the same revascularisation algorithm in this study limits the validity of the simulated vasculature. Future investigations should include a more rigorous approach to acquire sufficient data to provide boundary, initial and final conditions of the vasculature.

In conclusion, we present a model of bone regeneration that encompasses bone formation, differentiation, and tissue resorption across all phases of bone regeneration. The simulated changes in bone volume fraction over time were validated against an *in vivo* osteotomy model and predicted regeneration outcomes given different sizes of simulated osteotomy gaps with a maximum of 5.5% RMSE. The micro-MPA model can contribute to collective initiatives aimed at advancing the 3R principles (reduce, refine, replace) and steering forthcoming experimental investigations on bone repair. Experimental proposals, such as those exploring early mechanical loading of conservatively treated fractures or elucidating non-union mechanisms, could benefit from initial scrutiny using the micro-MPA model. This preliminary evaluation would help identify critical examination timepoints and assess the overall feasibility of testing central hypotheses before progressing to *in vivo* experimentation. In this manner, the micro-MPA model constitutes a new evaluative tool that can accelerate research in bone regeneration.

## Data Availability

The raw data supporting the conclusions of this article will be made available by the authors, without undue reservation.
