# Supplementary material for: An in silico micro-multiphysics agent-based approach for simulating bone regeneration in a mouse femur defect model
Source: Front Bioeng Biotechnol. 2023 Dec 14;11:1289127. doi: 10.3389/fbioe.2023.1289127 (PMC10757951; doi:10.3389/fbioe.2023.1289127)
Supplement: Supplementary file 6 [file DataSheet1.docx]

Supplementary Material

An *In Silico* Micro-Multiphysics Agent-Based Approach for Simulating Bone Regeneration in a Mouse Femur Defect Model

**Jack J. Kendall^1,2^, Charles Ledoux^1^, Francisco C. Marques^1^, Daniele Boaretti^1^, Friederike A. Schulte^1^, Elise F. Morgan^2^, Ralph Müller^*1^**

^1^Institute for Biomechanics, ETH Zurich, Zurich, Switzerland

^2^Center for Multiscale and Translational Mechanobiology, Boston University, Boston, MA, USA

*** Correspondence:** Ralph Müller, ram@ethz.ch

# Abbreviations

| **Abbreviation** | **Description** |
| --- | --- |
| ABM | Agent-based model |
| ANOVA | Analysis of variance |
| BFR | Bone formation rate |
| BRR | Bone resorption rate |
| BV/TV | Bone volume fraction |
| CSCS | Swiss National Supercomputing Centre |
| DC | Defect centre |
| DP | Defect periphery |
| dt | Timestep |
| ECM | Extracellular matrix |
| EFF | Effective strain |
| FC | Fragment centre |
| FP | Fragment periphery |
| FQR | Formation, quiescence and resorption |
| $\boldsymbol{f}_{VEGF}$ | VEGF production function |
| HSC | Hematopoietic stem cell |
| LRP5/6 | Lipoprotein receptor-related protein 5/6 |
| mg HA/cm^3^ | Milligrams of hydroxyapatite per cubic centimetre |
| micro-CT | Micro-computed tomography |
| micro-FE | Micro-finite element analysis |
| micro-MPA | Micro-multiphysics agent-based |
| n | Number of samples |
| ParOSol | Parallel Octree Solver |
| POD | Post-operative day |
| OBL | Osteoblast |
| OCL | Osteoclast |
| OG | Osteotomy gap |
| OPG | Osteoprotegerin |
| RANK | Receptor activator of nuclear factor *κ*β |
| RANKL | Receptor activator of nuclear factor *κ*β ligand |
| RDD | Reaction, Diffusion, Decay |
| RMSE | Root mean squared error |
| Scl | Sclerostin |
| SED | Strain energy density |
| TGF-β1 | Transforming growth factor beta 1 |
| TMD | Tissue mineral density |
| TV | Total volume |
| VEGF | Vascular endothelial growth factor |
| VOI | Volume of interest |
| VV | Vascular volume |
| ε_FOR_ | Osteogenic strain threshold |
| ε_RES_ | Osteolytic strain threshold |

# Supplementary Data

## Mineralisation

While osteoblasts synthesised osteoid, the mineralisation of the osteoid was determined to be largely independent of cellular behaviour. The extracellular fluid is saturated with hydroxyapatite which condenses and crystallises between collagen fibres (Nair et al., 2013; Ping et al., 2022). Osteoblasts have been proposed to enable mineralisation by secreting vesicles containing hydroxyapatite nano-particles, which could act as a source of nuclei for precipitation of mineral (Boonrungsiman et al., 2012). The local mineral concentration $C_{mineral}$ at position x was determined in the micro-MPA model by the mineralisation of osteoid and the removal via osteoclasts which was represented as $S_{mineral}(x)$. The mineralisation was a passive process whereby the given osteoid concentration $C_{osteoid}(x)$ within a voxel presented the available volume for mineral to form:

$$\frac{\Delta C_{mineral}(x)}{\Delta t}=r_{mineral}\left( C_{osteoid}(x)-C_{mineral}(x) \right)\cdot C_{mineral}^{\left( aq \right)}-S_{mineral}(x)$$

The speed of mineralisation was determined by the available organic matrix (difference in concentration between osteoid and mineral) and the mineralisation rate $r_{mineral}$. The extracellular fluid was assumed to be fully saturated with mineral, such that $C_{mineral}^{\left( aq \right)}=1$. The mineralisation was calculated with every cell update (dt_mineral = dt_cells).

# Supplementary Figure and Tables

## Supplementary Figures


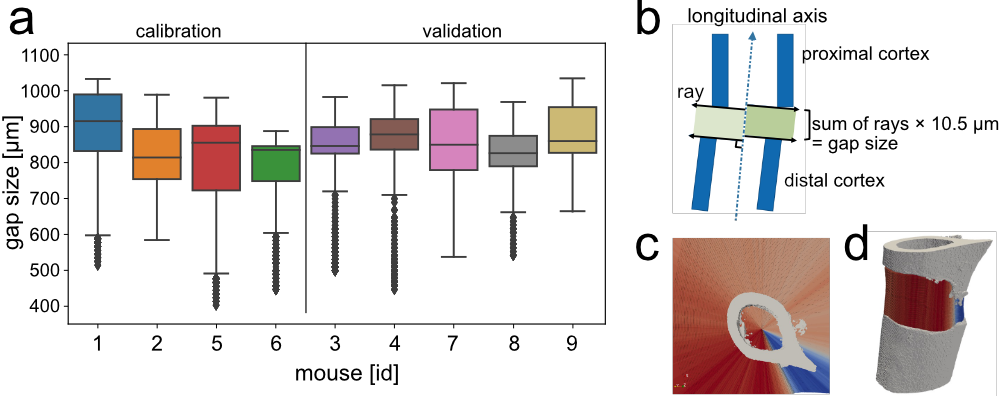


Supplementary Figure 1 (a) Osteotomy gap sizes for the different mice which were randomly assigned to either the calibration or validation dataset. Mouse 5 was used for the *in silico* study of the osteotomy gap sizes. (b) The gap sizes were measured via raytracing along the longitudinal axis of the femur between the two cortical fragments. The gap width was determined by counting the number of rays perpendicular to the longitudinal axis which fit between the cortices – similar to measurements taken with calipers – and multiplying with the voxel height. (c) The procedure was repeated until all widths across the entire gap were mapped. (d) The distances were then masked using the defect’s cortical region and skeletonised.


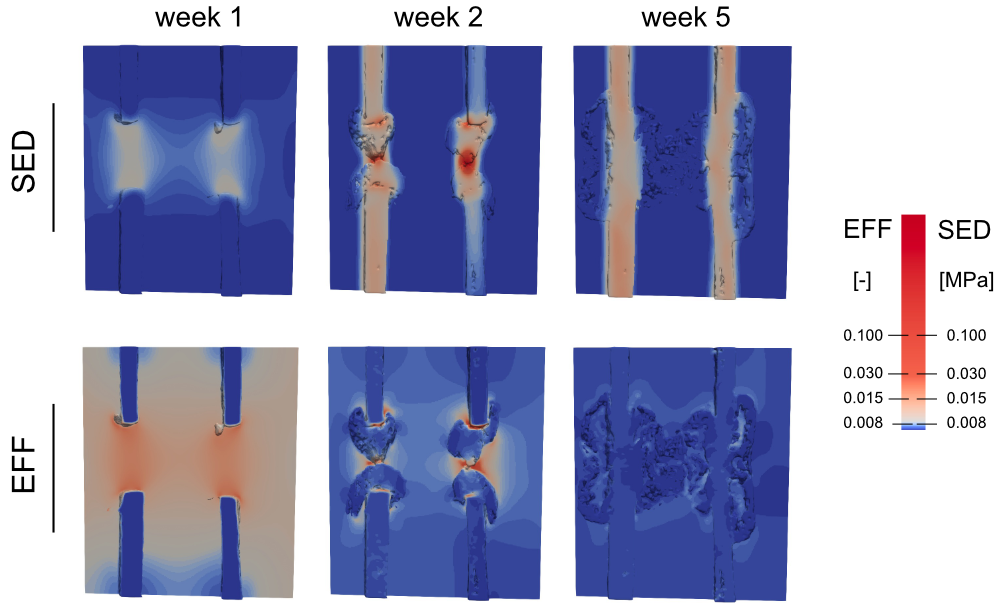


Supplementary Figure 2 Renderings of the predicted EFF and SED mechanical stimuli in mineralized and soft tissue for the representative mouse 7 at week 1, 2, and 5. The mineral structures were thresholded at 720 mg HA/cm^3^.


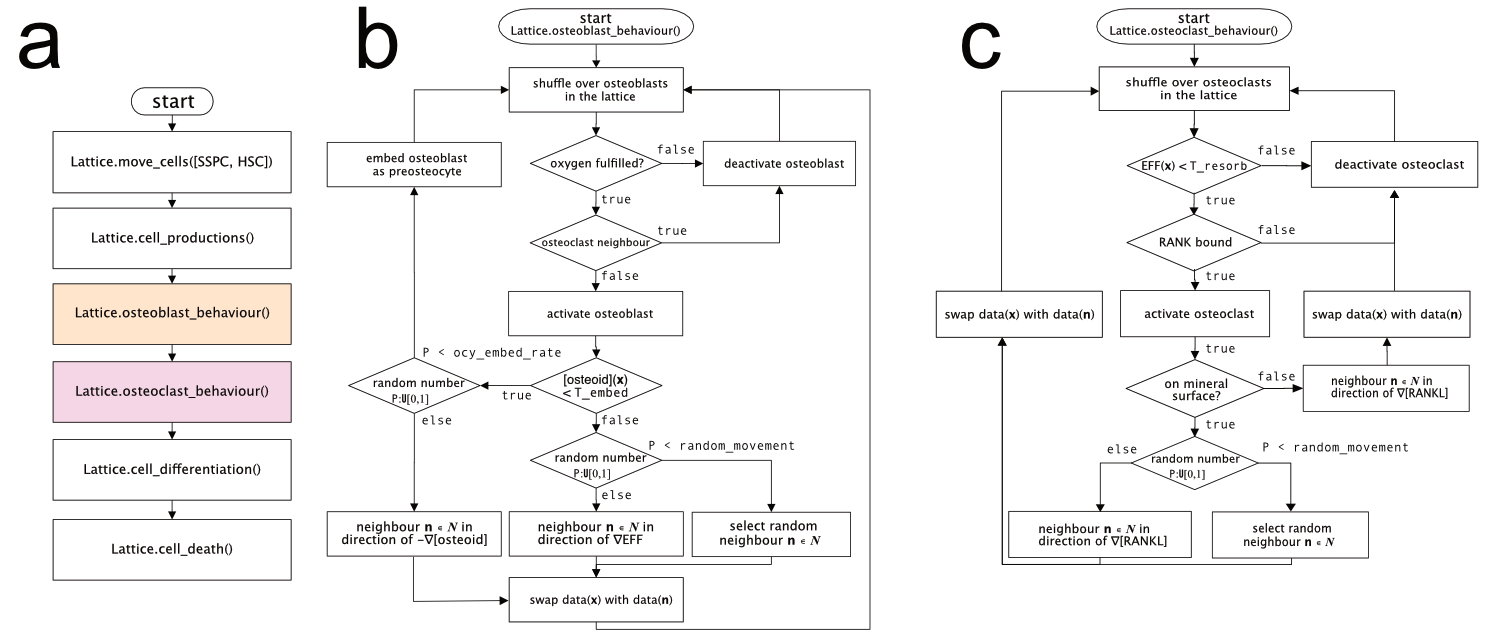


Supplementary Figure 3 (a) The time step of the cells is executed in the following order: Move Cells; Cell cytokine and ECM production; Osteoblast Behaviour (b); Osteoclast Behaviour (c); Cell Differentiation and Proliferation; Cell Death.


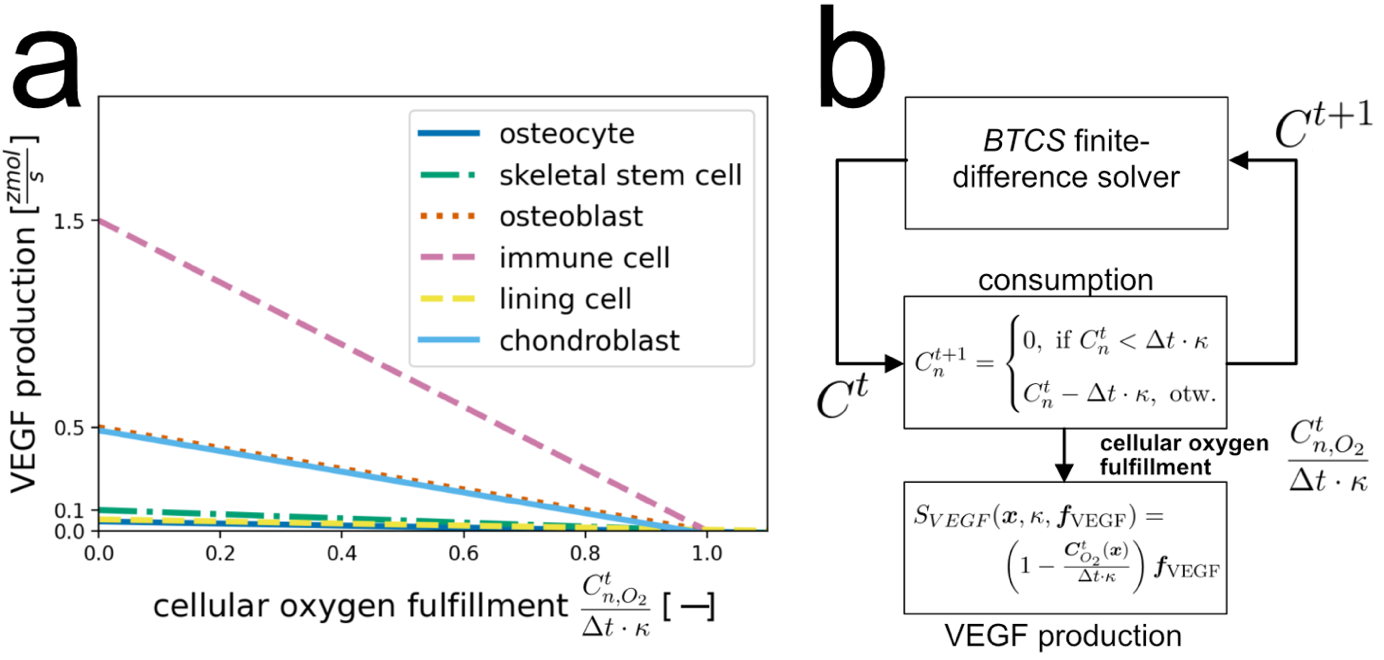


Supplementary Figure 4 (a) VEGF activation functions link a cell’s oxygen fulfilment to its VEGF production. Lining cells and osteocytes have a maximum VEGF production rate of 0.05 zmol/s. (b) Schematic of the oxygen consumption and the update with the backward time centered space (BTCS) finite-difference solver. The oxygen concentration at timepoint t+1 (Ct+1) is calculated using the consumption rate (𝜅) and the time step (dt) at timepoint t. The VEGF production of each cell $\boldsymbol{f}_{\boldsymbol{VEGF}}$

is then a function of the cellular oxygen fulfilment (see a).


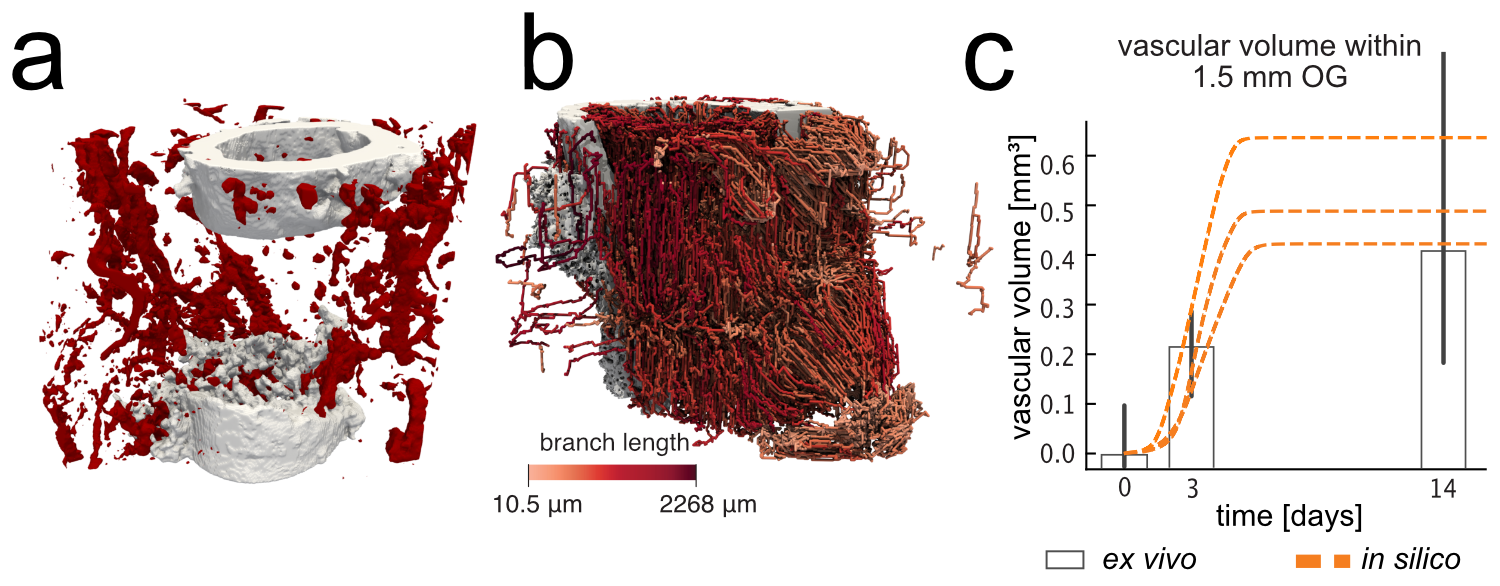


Supplementary Figure 5 (a) Rendering of the mineral and vascular tissue *ex vivo* micro-CT data retrieved from Morgan et al. (2012) at POD 14. (b) Rendering of the *in silico* vascular volume where the colours are mapped to represent vessel length. (c) Predicted vascular volume for three 1.5 mm osteotomies (Morgan et al., 2012) was plotted against the measured vascular volume for POD 0, 3, and 14. Adapted from Kendall et al. (2022).


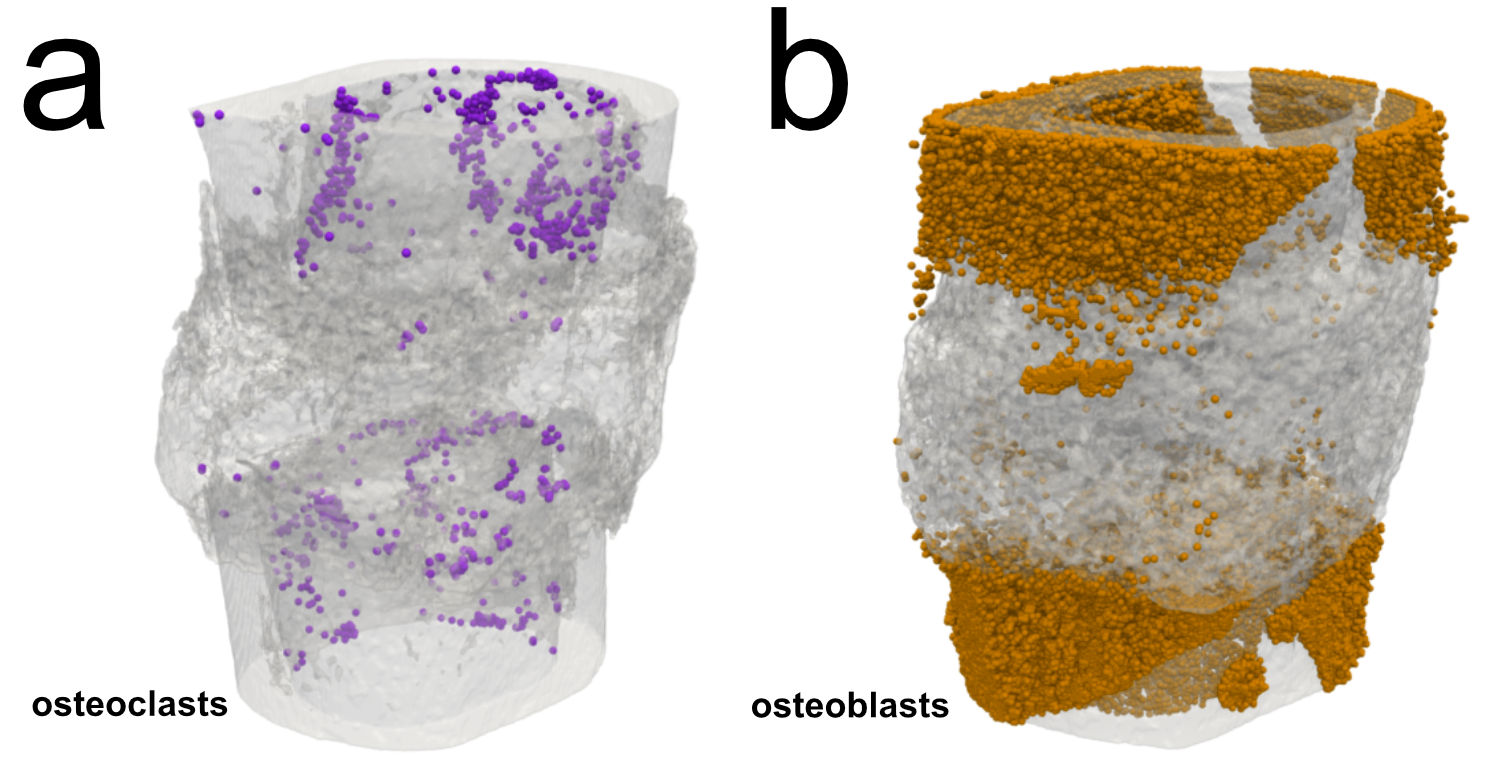


Supplementary Figure 6 Clustering of (a) osteoclasts after POD 14 and (b) osteoblasts after POD 35.

## Supplementary Tables

**Table S1:** Parameters of the *in silico* model.

| **Parameter** | **Description** | **Value** | **Reference** |
| --- | --- | --- | --- |
| $D_{cytokines, tissue}$ | Diffusivity of cytokines in soft tissue | $1.0\times{10}^{-4} {mm}^{2}s^{-1}$ | (Yu et al., 2009) |
| $D_{cytokines, Bone}$ | Diffusivity of cytokines in bone | $1.0\times{10}^{-6} {mm}^{2}s^{-1}$ | Estimated from (Fernández-Seara et al., 2002) |
| $D_{oxygen}$ | Diffusivity of oxygen | $2.2\times{10}^{-4} {mm}^{2}s^{-1}$ | (Yoshida and Ohshima, 1966) |
| $\lambda^{TGF-beta}$ | Decay of TGF-beta | $16\times{10}^{-4} s^{-1}$ | (Wakefield et al., 1990) |
| $\lambda$ | Decay of all the other cytokines | $3.8\times{10}^{-4} s^{-1}$ | (Eppler et al., 2002) |
| $k_{LRP5/6,Scl}^{f}$ | Forward binding coefficient for LRP5/6-Scl | $0.95\times{10}^{-1} 1/(zmol s)$ | (Boaretti et al., 2023) |
| $k_{LRP5/6,Scl}^{r}$ | Backward binding coefficient for LRP5/6-Scl | $1.0\times{10}^{-4} s^{-1}$ |  |
| $k_{TGF-beta\_rec,TGF-beta}^{f}$ | Forward binding coefficient for TGF-beta_rec- TGF-beta | $2.0\times{10}^{-2} 1/(zmol s)$ |  |
| $k_{TGF-beta\_rec,TGF-beta}^{r}$ | Backward binding coefficient for TGF-beta_rec- TGF-beta | $1.0 \times{10}^{-3} s^{-1}$ |  |
| $k_{\mathrm{VEGFR},VEGF}^{f}$ | Forward binding coefficient for VEGFR- VEGF | $2.0\times{10}^{-2} 1/(zmol s)$ | (Kendall et al., 2022) |
| $k_{\mathrm{VEGFR},VEGF}^{r}$ | Backward binding coefficient for VEGFR- VEGF | $1.0 \times{10}^{-3} s^{-1}$ | (Kendall et al., 2022) |
| $k_{RANK,RANKL}^{f}$ | Forward binding coefficient for RANK-RANKL | $4.0\times{10}^{-1} 1/(zmol s)$ | (Boaretti et al., 2023) |
| $k_{RANK,RANKL}^{r}$ | Backward binding coefficient for RANK-RANKL | $1.0 \times{10}^{-3} s^{-1}$ |  |
| $k_{OPG,RANKL}^{f}$ | Forward binding coefficient for OPG-RANKL | $1.0\times{10}^{-1} 1/(zmol s)$ |  |
| $k_{OPG,RANKL}^{r}$ | Backward binding coefficient for OPG-RANKL | $8.0\times{10}^{-4} s^{-1}$ |  |
| $\omega_{OBL}^{EFF}$ | Osteoblast biased movement probability towards EFF | 0.4 | Calibrated to (Wehrle et al., 2019) |
| $\omega_{OCL}^{RANKL}$ | Osteoclast biased movement probability towards RANKL | 0.4 | Calibrated to (Wehrle et al., 2019) |
| $\omega_{cells}$ | Random movement probability | 0.2 | (Appeddu and Shur, 1994) |
| $\omega_{tip cell}^{VEGF}$ | Tip cell biased movement probability towards VEGF | 0.7 | (Checa and Prendergast, 2009) |
| $\omega_{tip cell}$ | Tip cell random movement probability | 0.15 | (Checa and Prendergast, 2009) |
| $\tau^{VEGF}$ | VEGF bound receptor threshold: tip cell activation if > $\tau$ | 0.5 | (Kendall et al., 2022) |
| $\tau^{LRP5/6}$ | LRP5/6 bound receptor threshold: SSPC and OBL differentiate into lining cells if > $\tau$ | 0.7 | Calibrated to (Wehrle et al., 2019) |
| $\tau^{RANK}$ | RANK bound receptor threshold: HSC differentiate into osteoclasts if > $\tau$ | 0.5 | Calibrated to (Wehrle et al., 2019) |
| $\tau^{TGF-beta}$ | TGF-beta bound receptor threshold: SSPC and OBL proliferation scale increased by factor  $2$ if > $\tau$, also SSPC differentiation to OBL | 0.6 | Calibrated to (Wehrle et al., 2019) |
| $\sigma_{OBL}$ | Osteoblast polarisation (std. dev., mean=0) | 0.45 | (Tourolle, 2019) |
| $\sigma_{OCL}$ | Osteoclast polarisation (std. dev., mean=0) | 0.25 | (Tourolle, 2019) |
| $r^{mineral}$ | Mineralisation rate | 0.121 ${day}^{-1}$ | Calibrated to (Wehrle et al., 2019) |
| $\beta_{osteocyte}^{Scl}$ | Max osteocyte anti-anabolic production rate of Scl | $1.0\times{10}^{-3}zmol /20 min$ | (Boaretti et al., 2023) |
| $\beta_{osteocyte}^{RANKL}$ | Max osteocyte catabolic production rate of RANKL | $0.25\times{10}^{-2}zmol /20 min$ | (Boaretti et al., 2023) |
| $\beta_{osteocyte}^{OPG}$ | Max osteocyte anti-catabolic production rate of OPG | $0.3\times{10}^{-2}zmol /20 min$ | (Boaretti et al., 2023) |
| $P_{SSPC}$ | SSPC proliferation rate | $0.04 {day}^{-1}$ | (Tourolle, 2019) |
| $P_{HSC}$ | HSC proliferation rate | $0.08 {day}^{-1}$ |  |
| $P_{OBL}$ | Osteoblast proliferation rate | $0.01 {day}^{-1}$ |  |
| $A_{osteocyte}$ | Osteocyte apoptosis rate | $6.8\times{10}^{-5} {day}^{-1}$ |  |
| $A_{OBL}$ | Osteoblast apoptosis rate | $0.01 {day}^{-1}$ |  |
| $A_{OCL}$ | Osteoclast apoptosis rate | $0.5\times{10}^{-2} {day}^{-1}$ |  |
| $\beta_{OCL}^{mineral, osteoid}$ | Max osteoclast resorption rate of mineral and osteoid | $20 mg HA/day$ | (Repp et al., 2015) |
| $\beta_{OBL}^{osteoid}$ | Max osteoblast synthesis rate of osteoid | $120 eqv. mg HA/day$ | Calibrated to (Wehrle et al., 2019) |
| $\beta_{OBL, Li.Ce}^{RANKL}$ | Max osteoblast and lining cell catabolic production rate of RANKL | $0.4\times{10}^{-3}zmol /20 min$ | (Boaretti et al., 2023) |
| $\beta_{OBL, Li.Ce}^{OPG}$ | Max osteoblast and lining cell anabolic production rate of OPG | $0.6\times{10}^{-2}zmol /20 min$ | (Boaretti et al., 2023) |
| dt_cells_ | Timestep for cells and vasculature | 20 minutes | (Appeddu and Shur, 1994) |
| dt_RDD_ | Timestep for reaction-diffusion-decay of the cytokines | 30 minutes | Given dt_cells_, Maximum timestep |
| dt_micro-FE_ | Timestep for update of the mechanical signal | 8 hours | (Tourolle, 2019) |
| $k$ | Oxygen consumption | $2.042 fmol /s$ | Calibrated to (Brighton and Krebs, 1972; Epari et al., 2008) |
| $\varepsilon_{FOR}$ | Mechanical signal threshold for osteogenic pathways | 0.008 MPa or 0.008 | (Razi et al., 2015) |
| $\varepsilon_{RES}$ | Mechanical signal threshold for osteolytic pathways | 0.015 MPa or 0.015 | (Razi et al., 2015) |
| $\tau_{OBL}^{mineral}$ | OBL embedding threshold: OBL differentiate to pre-osteocytes if mineral > $\tau$ | $180 mg HA/{cm}^{3}$ | Calibrated to (Mader et al., 2013) |
| $r^{osteocytes}$ | Pre-Osteocyte to osteocyte embedding probability | 0.1 | Calibrated to (Mader et al., 2013) |

**Table S2:** Initial concentration values for all chemical species in the micro-MPA model.

| **Molecule** | **Initial Concentration (POD 0)** | | |
| --- | --- | --- | --- |
|  | **VOI** | **Value**  $\boldsymbol{\mu mol/}\boldsymbol{m}^{\boldsymbol{3}}$ | **Reference** |
| RANKL | Mineral | $15.99\cdot{10}^{4}$ | (Shahnazari et al., 2012) |
|  | else | 0 | Estimated |
| OPG | Soft tissue | $4.5\cdot{10}^{4}$ | (Shahnazari et al., 2012) |
|  | else | 0 | Estimated |
| RANKL-OPG | All | 0 | Estimated |
| VEGF | DC | 0.00326 | (Condon et al., 2004) |
|  | else | 0.000652 | Estimated |
| Scl | DC + FC | $0.0114$ | (Shahnazari et al., 2012) |
|  | else | 0 | Estimated |
| TGF-beta | DC | 15.99 | (Wahl et al., 1990) |
|  | else | $15.99\cdot{10}^{-4}$ |  |
| Oxygen | DC | $0.325\cdot{10}^{6}$ | (Epari et al., 2008) |
|  | else | $0.065 \cdot{10}^{6}$ |  |

**Table S3:** Initial cell seeding densities for all cell types in the micro-MPA model.

| **Cell** | **Initial Cell Seeding (POD 0)** | | |
| --- | --- | --- | --- |
|  | **VOI** | **Density** | **Reference** |
| SSPC | Periosteum | $15 \cdot{10}^{6} {ml}^{-1}$ | (Tourolle, 2019) |
|  | Periosteal Surface (Cambium) | 20 % | (Moore et al., 2014) |
|  | Endosteum | $5 \cdot{10}^{5} {ml}^{-1}$ | (Galotto et al., 1999) |
|  | DC | $1 \cdot{10}^{6} {ml}^{-1}$ | (Tourolle, 2019) |
| Osteoblast | - | - | - |
| Lining cell | Periosteal Surface (Cambium) | 60 % | (Moore et al., 2014) |
|  | Endosteal Surface | 60 % | (Dedeepiya et al., 2012) |
| Pre-osteocyte | - | - | - |
| Osteocyte | Mineral | $45’000 {mm}^{-3}$ | (Mader et al., 2013) |
| HSC | FC | $5 \cdot{10}^{5} {ml}^{-1}$ | (Dedeepiya et al., 2012) |
| Osteoclast | - | - | - |
| Immune cell | DC | $12 \cdot{10}^{6} {ml}^{-1}$ | (O’Connell et al., 2015) |
| Tip cell | Periosteum + Endosteum | $110 {mm}^{-2}$ | (Morgan and Lei, 2015) |

# References

Appeddu, P. A., and Shur, B. D. (1994). Molecular analysis of cell surface beta-1,4-galactosyltransferase function during cell migration. *Proc. Natl. Acad. Sci.* 91, 2095–2099. doi: 10.1073/pnas.91.6.2095.

Boaretti, D., Marques, F. C., Ledoux, C., Singh, A., Kendall, J. J., Wehrle, E., et al. (2023). Trabecular bone remodellng in the aging mouse: A micro-multiphysics agent-based in silico model using single-cell mechanomics. *Front. Bioeng. Biotechnol.* 11, 1091294. doi: 10.3389/fbioe.2023.1091294.

Boonrungsiman, S., Gentleman, E., Carzaniga, R., Evans, N. D., McComb, D. W., Porter, A. E., et al. (2012). The role of intracellular calcium phosphate in osteoblast-mediated bone apatite formation. *Proc. Natl. Acad. Sci.* 109, 14170–14175. doi: 10.1073/pnas.1208916109.

Brighton, C. T., and Krebs, A. G. (1972). Oxygen tension of healing fractures in the rabbit. *J. Bone Joint Surg. Am.* 54, 323–332.

Checa, S., and Prendergast, P. J. (2009). A Mechanobiological Model for Tissue Differentiation that Includes Angiogenesis: A Lattice-Based Modeling Approach. *Ann. Biomed. Eng.* 37, 129–145. doi: 10.1007/s10439-008-9594-9.

Condon, E. T., Wang, J. H., and Redmond, H. P. (2004). Surgical injury induces the mobilization of endothelial progenitor cells. *Surgery* 135, 657–661. doi: 10.1016/j.surg.2003.10.012.

Dedeepiya, V. D., Rao, Y. Y., Jayakrishnan, G. A., Parthiban, J. K. B. C., Baskar, S., Manjunath, S. R., et al. (2012). Index of CD34+ Cells and Mononuclear Cells in the Bone Marrow of Spinal Cord Injury Patients of Different Age Groups: A Comparative Analysis. *Bone Marrow Res.* 2012, 1–8. doi: 10.1155/2012/787414.

Epari, D. R., Lienau, J., Schell, H., Witt, F., and Duda, G. N. (2008). Pressure, oxygen tension and temperature in the periosteal callus during bone healing—An in vivo study in sheep. *Bone* 43, 734–739. doi: 10.1016/j.bone.2008.06.007.

Eppler, S. M., Combs, D. L., Henry, T. D., Lopez, J. J., Ellis, S. G., Yi, J.-H., et al. (2002). A target-mediated model to describe the pharmacokinetics and hemodynamic effects of recombinant human vascular endothelial growth factor in humans*. *Clin. Pharmacol. Ther.* 72, 20–32. doi: 10.1067/mcp.2002.126179.

Fernández-Seara, M. A., Wehrli, S. L., and Wehrli, F. W. (2002). Diffusion of Exchangeable Water in Cortical Bone Studied by Nuclear Magnetic Resonance. *Biophys. J.* 82, 522–529. doi: 10.1016/S0006-3495(02)75417-9.

Galotto, M., Berisso, G., Delfino, L., Podesta, M., Ottaggio, L., Dallorso, S., et al. (1999). Stromal damage as consequence of high-dose chemo/radiotherapy in bone marrow transplant recipients. *Exp. Hematol.* 27, 1460–1466. doi: 10.1016/S0301-472X(99)00076-4.

Kendall, J. J., Tourolle, D. C., Boaretti, D., Marques, F. C., Morgan, E. F., and Müller, R (2022). Micro-multiphysics agent-based modeling of revascularization during bone healing. in *Trans. Orthop. Res. Soc.*, 474. Available at: https://www.ors.org/transactions/68/474.pdf.

Mader, K. S., Schneider, P., Müller, R., and Stampanoni, M. (2013). A quantitative framework for the 3D characterization of the osteocyte lacunar system. *Bone* 57, 142–154. doi: 10.1016/j.bone.2013.06.026.

Moore, S. R., Milz, S., and Knothe Tate, M. L. (2014). Periosteal thickness and cellularity in mid-diaphyseal cross-sections from human femora and tibiae of aged donors. *J. Anat.* 224, 142–149. doi: 10.1111/joa.12133.

Morgan, E. F., Hussein, A. I., Al-Awadhi, B. A., Hogan, D. E., Matsubara, H., Al-Alq, Z., et al. (2012). Vascular development during distraction osteogenesis proceeds by sequential intramuscular arteriogenesis followed by intraosteal angiogenesis. *Bone* 51, 535–545. doi: 10.1016/j.bone.2012.05.008.

Morgan, E. F., and Lei, J. (2015). Toward Clinical Application and Molecular Understanding of the Mechanobiology of Bone Healing. *Clin. Rev. Bone Miner. Metab.* 13, 256–265. doi: 10.1007/s12018-015-9197-6.

Nair, A. K., Gautieri, A., Chang, S.-W., and Buehler, M. J. (2013). Molecular mechanics of mineralized collagen fibrils in bone. *Nat. Commun.* 4, 1724. doi: 10.1038/ncomms2720.

O’Connell, K. E., Mikkola, A. M., Stepanek, A. M., Vernet, A., Hall, C. D., Sun, C. C., et al. (2015). Practical murine hematopathology: a comparative review and implications for research. *Comp. Med.* 65, 96–113.

Ping, H., Wagermaier, W., Horbelt, N., Scoppola, E., Li, C., Werner, P., et al. (2022). Mineralization generates megapascal contractile stresses in collagen fibrils. *Science* 376, 188–192. doi: 10.1126/science.abm2664.

Razi, H., Birkhold, A. I., Weinkamer, R., Duda, G. N., Willie, B. M., and Checa, S. (2015). Aging Leads to a Dysregulation in Mechanically Driven Bone Formation and Resorption: MECHANOREGULATION OF (RE)MODELING. *J. Bone Miner. Res.* 30, 1864–1873. doi: 10.1002/jbmr.2528.

Repp, F., Vetter, A., Duda, G. N., and Weinkamer, R. (2015). The connection between cellular mechanoregulation and tissue patterns during bone healing. *Med. Biol. Eng. Comput.* 53, 829–842. doi: 10.1007/s11517-015-1285-8.

Shahnazari, M., Dwyer, D., Chu, V., Asuncion, F., Stolina, M., Ominsky, M., et al. (2012). Bone turnover markers in peripheral blood and marrow plasma reflect trabecular bone loss but not endocortical expansion in aging mice. *Bone* 50, 628–637. doi: 10.1016/j.bone.2011.11.010.

Tourolle, D. (2019). A micro-scale multiphysics framework for fracture healing and bone remodelling. 186 p. doi: 10.3929/ETHZ-B-000364637.

Wahl, S. M., McCARTNEY-FRANCIS, N., Allen, J. B., Dougherty, E. B., and Dougherty, S. F. (1990). Macrophage Production of TGF-? and Regulation by TGF-? *Ann. N. Y. Acad. Sci.* 593, 188–196. doi: 10.1111/j.1749-6632.1990.tb16111.x.

Wakefield, L. M., Winokur, T. S., Hollands, R. S., Christopherson, K., Levinson, A. D., and Sporn, M. B. (1990). Recombinant latent transforming growth factor beta 1 has a longer plasma half-life in rats than active transforming growth factor beta 1, and a different tissue distribution. *J. Clin. Invest.* 86, 1976–1984. doi: 10.1172/JCI114932.

Wehrle, E., Tourolle né Betts, D. C., Kuhn, G. A., Scheuren, A. C., Hofmann, S., and Müller, R. (2019). Evaluation of longitudinal time-lapsed in vivo micro-CT for monitoring fracture healing in mouse femur defect models. *Sci. Rep.* 9, 17445. doi: 10.1038/s41598-019-53822-x.

Yoshida, F., and Ohshima, N. (1966). Diffusivity of oxygen in blood serum. *J. Appl. Physiol.* 21, 915–919. doi: 10.1152/jappl.1966.21.3.915.

Yu, S. R., Burkhardt, M., Nowak, M., Ries, J., Petrášek, Z., Scholpp, S., et al. (2009). Fgf8 morphogen gradient forms by a source-sink mechanism with freely diffusing molecules. *Nature* 461, 533–536. doi: 10.1038/nature08391.

# 
